# Orthogonal photochemistry-assisted printing of 3D tough and stretchable conductive hydrogels

**DOI:** 10.1038/s41467-021-21869-y

**Published:** 2021-04-07

**Authors:** Hongqiu Wei, Ming Lei, Ping Zhang, Jinsong Leng, Zijian Zheng, You Yu

**Affiliations:** 1 Key Laboratory of Synthetic and Natural Functional Molecule Chemistry of the Ministry of Education, College of Chemistry and Materials Science, Northwest University, Xi’an, China; 2 School of Astronautics, Northwestern Polytechnical University, Xi’an, China; 3 Center for Composite Materials and Structures, Harbin Institute of Technology, Harbin, China; 4 Institute of Textiles and Clothing & Research Institute for Smart Energy, The Hong Kong Polytechnic University, Hong Kong, China

**Keywords:** Electronic materials, Conjugated polymers, Gels and hydrogels, Composites

## Abstract

3D-printing tough conductive hydrogels (TCHs) with complex structures is still a challenging task in related fields due to their inherent contrasting multinetworks, uncontrollable and slow polymerization of conductive components. Here we report an orthogonal photochemistry-assisted printing (OPAP) strategy to make 3D TCHs in one-pot via the combination of rational visible-light-chemistry design and reliable extrusion printing technique. This orthogonal chemistry is rapid, controllable, and simultaneously achieve the photopolymerization of EDOT and phenol-coupling reaction, leading to the construction of tough hydrogels in a short time (*t*_gel_ ~30 s). As-prepared TCHs are tough, conductive, stretchable, and anti-freezing. This template-free 3D printing can process TCHs to arbitrary structures during the fabrication process. To further demonstrate the merits of this simple OPAP strategy and TCHs, 3D-printed TCHs hydrogel arrays and helical lines, as proofs-of-concept, are made to assemble high-performance pressure sensors and a temperature-responsive actuator. It is anticipated that this one-pot rapid, controllable OPAP strategy opens new horizons to tough hydrogels.

## Abstract (web summary)

3D-printing tough conductive hydrogels (TCHs) with complex structures is still a challenging task due to their inherent contrasting multinetworks, uncontrollable and slow polymerization. Here the authors show an orthogonal photochemistry-assisted printing strategy to make 3D TCHs in one pot.

## Introduction

Tough conductive hydrogels (TCHs) have been attracting much interest over the past decade because of their excellent conductivities, mechanical properties, and water-rich nature^1–6^. They have contrasting multinetworks that give them high mechanical strength and the ability to efficiently dissipate mechanical energy via different dissipation mechanisms, leading to withstanding strains to maintain integrity. Such hydrogels have a wide range of applications, e.g., in electronics, tissue engineering, actuators, and energy-storage devices^7–14^. Typical conductive polymers such as polyaniline (PANI), poly(3,4-ethylenedioxythiophene) (PEDOT), and polypyrrole (PPy) are critical components of TCHs^15–19^. These polymers are generally introduced into hydrogels by directly soaking or mixing them with other precursors and in situ chemical oxidation or electrochemical polymerization of the monomers in the hydrogels^20–24^. Recently, strategies such as printing commercial conducting products, selective polymerizations, and electrochemical release of metal-ion crosslinkers have been reported for fabricating TCH patterns and 3D structures for applications, e.g., in vivo single-unit recording, strain sensors, and systematic transfer of electrical stimuli to encapsulated cells to enhance differentiation^25–29^. These pioneering studies have significantly broadened the applications of hydrogels to soft, wearable devices, and bioelectronics. However, the design of straightforward and rapid strategies for one-pot preparation of bulk TCHs and high-resolution patterns (~100 μm), especially with reported printing techniques for making complicated 3D structures is still a challenge.

In typical 3D extrusion printing techniques, inks can be extruded on-demand and then processed to give pre-designed architectures via fast solidification^30–33^. Visible-light-induced photogelation is an ideal method for preparing such 3D hydrogels because it has excellent biocompatibility and enables easy spatial control^34–36^. Light at long wavelengths (>400 nm) is recognized as safer triggers and has lower potential harm to active materials of cells, proteins, and DNA when comparing to these high-energy light sources (UV light or γ rays). Moreover, gelation processes can be initiated and then stopped by simply regulating irradiation procedures. However, some critical issues need to be considered when printing TCHs. For example, achieving uniform distributions of rigid PANI, PEDOT, and PPy in aqueous printing inks is challenging. Their deep colors may have adverse effects on the fabrication and mechanical properties of hydrogels: Visible-light absorption by conductive polymers is high, and this leads to low solidification efficiency and poor-quality 3D-structured TCHs. The monomers of these conductive polymers are relatively colorless and dissolve easily in printing inks, and therefore are better candidates for 3D printing of TCHs^37^. However, classic polymerization approaches are inefficient, uncontrollable, and lack broad compatibility to independently trigger other photoreactions for rapidly solidifying inks during the printing process (<40 s)^38,39^. A general, rapid, and controllable strategy for one-pot 3D printing of TCHs is, therefore, an intriguing target in this field.

In this work, we develop a rapid and controllable orthogonal photochemistry-assisted printing (OPAP) method for fabricating 3D TCHs. The model monomer (i.e., EDOT) is dissolved in aqueous ink and then extruded with tyramine-modified poly(vinyl alcohol) (PVA-Ph). Under visible-light irradiation, the ejected precursor is solidified in ~30 s, and the whole fabrication process was achieved in several tens of minutes. The freezing-treated TCHs are tough (2.25 MJ m^−3^), conductive (~2.0 S m^−1^), and have anti-freezing properties (~−36 °C), and can be stretched up to a maximum strain of 550% at critical stress of 0.8 MPa. Importantly, the high efficiency and controllability of the OPAP strategy make it suitable for printing complex 3D structures during the fabrication process. We use this strategy to produce 3D-printed TCH arrays and shape-memory helical lines for assembling flexible sensors and actuators, respectively, with high responsiveness to environmental pressure and temperature. This is a simple, time-saving, and straightforward OPAP strategy for rapidly printing high-performance 3D TCHs at mild conditions (Supplementary Table 1).

## Results

### Construction of tough conductive hydrogels via visible-light orthogonal chemistry

**Fig. 1:**
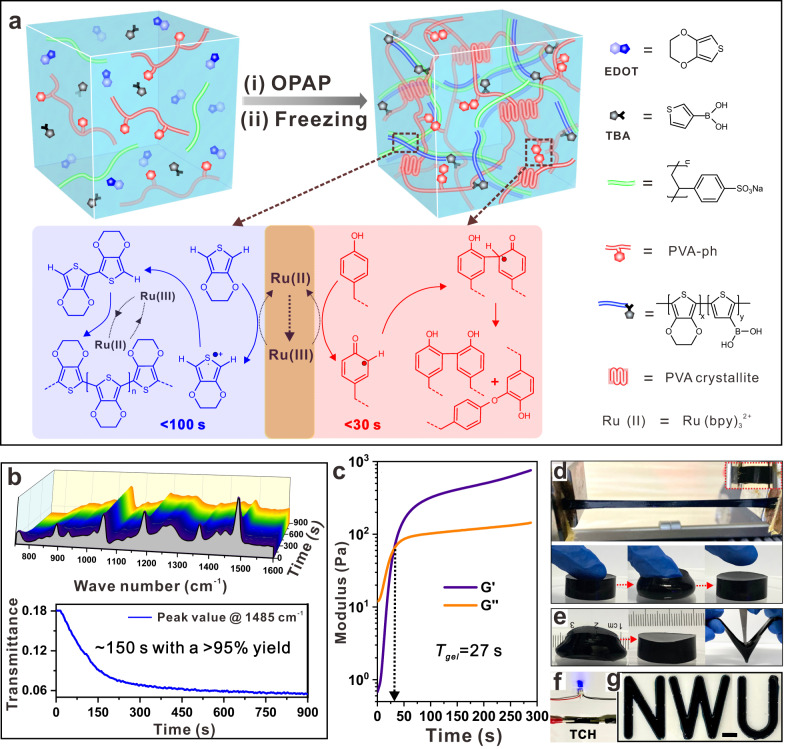
Construction of TCHs via visible-light orthogonal photochemistry design. **a** Independent chemical reactions of photopolymerization of PEDOT and coupling reaction of phenols with the catalysis of Ru(II)/APS. **b** Real-time FT-IR characterization of the polymerization of EDOT ([Ru(II)] = 0.33 mM, [APS] = 110 mM, [EDOT] = 46.5 mM) and **c** rheology measurement of PVA-Ph solution with increasing irradiation time. **d** Mechanical property and **e** toughness tests of as-prepared TCHs, respectively. **f** The digital images of a TCH ribbon powering a commercial LED light. **g** TCH letters of “N, W, U” were prepared in corresponding molds. The scale bar is 1 cm. All TCHs were prepared at the following condition: [Ru(II)] = 0.33 mM, [APS] = 110 mM, [PVA-Ph] = 10 wt% with the phenol content of 1%, [EDOT] = 93 mM, [TBA] = 3.7 mM, [PSS] = 2 wt%, and TEG/H_2_O = 2/3.

In terms of materials chemistry, the key factor in the OPAP strategy is the use of highly efficient ruthenium photochemistry to trigger two orthogonal photoreactions, namely a phenol-coupling reaction and polymerization of the conductive polymer precursors. These rapid and controllable reactions can be readily used in one-pot 3D printing of TCHs. PEDOT, PPy, and PANI are synthesized via similar chemical oxidation polymerization processes. Therefore, EDOT is used as a model monomer for preparing TCHs with PVA-Ph. As shown in Fig. 1a, the hydrogel precursors were water, EDOT, PVA-Ph, 3-thiopheneboronic acid (TBA), Ru(bpy)_3_Cl_2_/ammonium persulfate [Ru(II)/APS], triethylene glycol (TEG), and poly(4-styrenesulfonate) (PSS). A small amount of *p*-toluenesulfonic acid was added to adjust the pH of the precursor solution to 1–2. On exposure of the precursors to blue light (452 nm), excited Ru(II) is oxidized to Ru(III) by APS. This intermediate product is unstable and is strongly oxidizing in the phenol coupling of PVA-Ph and polymerization of EDOT^40,41^. Although the same reactant, i.e., Ru(III), catalyzes these orthogonal photoreactions, the reaction rates clearly differ (Fig. 1b, c and Supplementary Fig. 1). This can be ascribed to the formation of different radicals in the first rate-determining step^42^. Fourier-transform infrared (FT-IR) spectroscopy indicated that photopolymerization of EDOT, with a >95% yield, was achieved within 150 s (Fig. 1b). The phenol-coupling reaction is too fast to be monitored spectroscopically^40^. A rapid sol–gel transition was observed when an aqueous solution of PVA-Ph (10 wt%) was subjected to visible-light irradiation. Moreover, this process can be traced by recording the change of storage modulus (*G*′) and loss modulus (*G*″) in the rheology characterization of TCHs. These two moduli quantitatively reveal the elasticity and viscosity of samples in real time. As shown in Fig. 1c, the *G*′ was smaller than *G*″ because the hydrogel precursor was in a liquid state. When exposing it to light, both of them rapidly increased, and then *G*′ was larger than *G*″ after several tens of seconds of irradiation since the liquid precursor transformed into a hydrogel state. Therefore, the irradiation time (*G*′ = *G*″) was defined as the gelation time (*t*_gel_) of hydrogels. As for this study, the *t*_gel_ of TCHs was ~27 s. On the basis of a comparison of the rates of these two photoreactions, we can reasonably speculate that a PVA hydrogel is first constructed, and then PEDOT is generated and immobilized in this porous network. The light-yellow precursor solution is converted to a dark-blue hydrogel. This rapid phenol-coupling reaction significantly accelerates TCH photogelation and has potential applications in the 3D printing of advanced architectures. Moreover, it was found that freezing post-treatment significantly enhanced the mechanical properties and toughness of PVA-based hydrogels due to the formation of more PVA crystallites at a lower temperature (Supplementary Fig. 2)^22^. Therefore, if not especially mentioned, all the bulk and 3D-printed TCH samples were stored at −22 °C for 30 min and then slowly warmed to room temperature before testing and use in subsequent experiments. Because of the multinetworks typically found in TCHs, including PVA-crystallites-crosslinked and PEDOT/PSS networks, and a phenol–phenol network (shown in Fig. 1a), the as-prepared hydrogels are stretchable, compressible, tough, conductive, and can resist puncture and cutting (Fig. 1d–f and Supplementary Movie 1). The first two physically crosslinked networks can efficiently dissipate mechanical energy, and the second crosslinked network can maintain its integrity after releasing strains^43^. Bulk TCHs can be easily engineered into different shapes by pouring the precursors into the corresponding molds before irradiation (Fig. 1g). The introduction of PEDOT improves the electrochemical performances of hydrogels compared with those of PVA-Ph hydrogels (Supplementary Fig. 3).

### Mechanism of TCHs formation

**Fig. 2:**
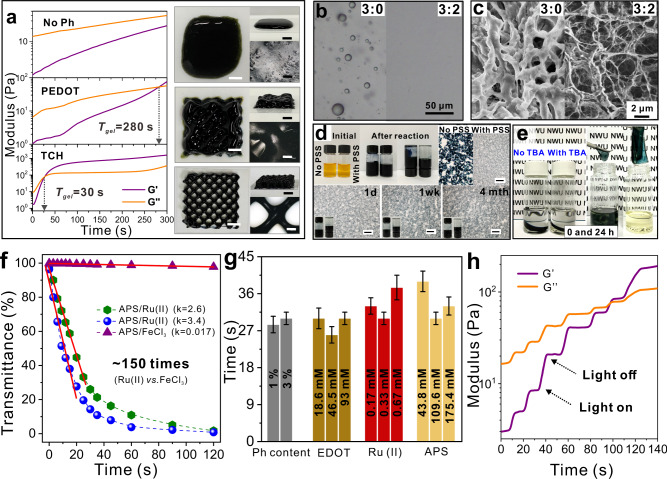
Effect of components on the formation of TCHs. **a** Real-time rheology characterizations of PVA-Ph, commercial PEDOT-containing PVA, and TCH, respectively. Digital images show products by extruding corresponding precursors and immediately exposing them to light irradiation. Scale bars are 1 cm. **b** Optical microscopy images of the TCH precursor and the control sample without TEG. **c** SEM images of as-prepared hydrogels using corresponding precursors in **b**. **d** Digital and microscopy images of precursors, as-prepared PEDOT/PSS solution, the control sample without the addition of PSS, and the solution with different storage time. Scale bars are 100 µm. **e** Digital images of TCHs and the control sample without TBA after immersing in DMSO for 48 h. **f** UV-vis transmittance of TCH precursors with increasing reaction time by using different catalysts. **g** Gelation time of TCHs with varying preparation conditions. Three samples were tested for each case. **h** Storage (*G*′) and loss (*G*″) modulus of TCHs with programmed intermittent light irradiation. TCHs were basically prepared at the following condition: [Ru(II)] = 0.33 mM, [APS] = 110 mM, [PVA-Ph] = 10 wt% with the phenol content of 1%, [EDOT] = 46.5 or 93 mM, [TBA] = 1.86 or 3.7 mM, [PSS] = 1 or 2 wt%, and TEG/H_2_O = 2/3. When evaluating the effect of one component on fabricating TCHs, other components were kept constant.

The design principle behind the OPAP strategy was clarified by systematically studying the effect of each component of this system on TCH fabrication. The effects of PVA-Ph and EDOT on the hydrogels were evaluated first because of their key roles in TCHs. Figure 2a and Supplementary Fig.  4a show that TCHs with a short *t*_gel_ of 30 s were rapidly prepared under visible-light irradiation. However, without phenol groups in the PVA chains, only EDOT photopolymerization occurred, and no sol–gel transition was observed even under lengthy irradiation (>300 s) (Supplementary Fig. 4b). Previous studies showed that directly introducing commercial PEDOT into tough hydrogels simplified fabrication procedures, and the steps for synthesizing this conductive polymer are not required^22,24,29,39^. But we found that dark-blue PEDOT strongly absorbed blue light and hindered its penetration into samples. The phenol-coupling rate of PVA-Ph was, therefore, considerably lower in this study. The sample was still in the liquid state after irradiation for 30 s (Supplementary Fig. 4c). The TCH *t*_gel_ was 280 s, which is about ten times that of the sample with EDOT monomers. Consequently, a precursor ink with a longer *t*_gel_ was not fully solidified during the fast printing process. The structure of pre-designed TCH meshes is challenging to be maintained as same as that prepared by the OPAP strategy (Fig. 2a, inset images). These results indicate that the use of PVA-Ph and EDOT monomers is necessary for TCH 3D printing.

EDOT is hydrophobic and its homogeneous dispersion in pure water is difficult. Figure 2b and Supplementary Fig. 5a show that emulsified monomer droplets of size <30 µm were present after vigorous stirring. After light irradiation, the resultant large PEDOT particles were encapsulated in brittle, dense hydrogels with poor mechanical properties (Fig. 2c and Supplementary Fig. 5b–d). The addition of TEG significantly enhanced the solubility of EDOT in this case, and a clear precursor solution was obtained with a TEG/H_2_O volume ratio of 2/3 (Supplementary Fig. 5a). No visible emulsion droplets of EDOT were formed, and the resultant PEDOT chains were more extended in the TCHs. Consequently, the as-prepared hydrogels were uniform, porous, and stretchable (Fig. 2b, c and Supplementary Fig. 5b–d). The role of PSS was clarified by using a specific solution without PVA-Ph to prepare PEDOT/PSS. Figure 2d shows that the as-prepared dark-blue solution was so stable that no sedimentation was observed even after more than 4 months. However, a microscopy image showed that many PEDOT particles were immediately aggregated on an ampere bottle in the control sample after light irradiation. This difference strongly indicates that PSS assists the homogeneous distribution of PEDOT in the TEG/H_2_O solution. Furthermore, we found that introduced difunctional TBAs can polymerize with EDOTs and covalently immobilize PEDOT chains on PVA networks via boronic acid ester chemistry (Supplementary Fig. 6). This unique structure helps maintain the TCH integrity in highly effective solvents such as water and DMSO (Fig. 2e and Supplementary Fig. 7).

Ru(II)/APS plays a crucial role in the fabrication of TCHs by the OPAP strategy. Figure 2f and Supplementary Fig. 8 show that under light irradiation, the transmittances of EDOT solutions at 550 nm in ultraviolet-visible (UV-vis) spectra sharply decreased to 0% within 30 s but remained at >95% after 120 s for a FeCl_3_/APS sample. Ru(II)/APS-catalyzed PEDOT polymerization more efficiently, and the polymerization rate was ~150 times that with the catalyst of FeCl_3_/APS at the same molar ratio. This was mainly attributed to the higher oxidation potential of Ru^3+/2+^ compared with that of Fe^3+/2+^ (1.24 vs. 0.7 V). A series of control experiments further indicated that a combination of Ru(II), APS, and the light was necessary for achieving EDOT photopolymerization (Supplementary Fig. 9). By varying the phenol content of PVA-Ph and concentrations of EDOT, Ru(II), and APS, we found that the *t*_gel_ values of TCHs can be tuned between 25 and 39 s, which was fast enough for 3D printing of TCHs (Fig. 2g and Supplementary Fig. 10)^34–36^. Moreover, real-time rheology and UV-vis spectroscopy were used to evaluate the controllability of this strategy for preparing 3D TCHs. Figure 2h shows that, as expected, the storage (*G*′) and loss (*G*″) moduli both immediately increased, but the transmittance decreased, with increasing irradiation time when the samples were exposed to intermittent visible-light irradiation (Supplementary Fig. 11). However, no change was observed in these two parameters when the sample was kept in the dark. These results strongly show that this orthogonal photochemistry is rapid and easily spatially controlled during the fabrication process. Note that although the catalyst of FeCl_3_/APS is extensively used to synthesize PEDOT at mild conditions, polymerization reactions spontaneously proceed, and reaction rates are difficult to be tuned by adjusting reaction conditions. Therefore, such a Ru(II)-catalyzed rapid and controllable process is particularly essential for applying this OPAP strategy to 3D printing of TCHs, as mentioned above.

### Mechanical properties and conductivity of TCHs

**Fig. 3:**
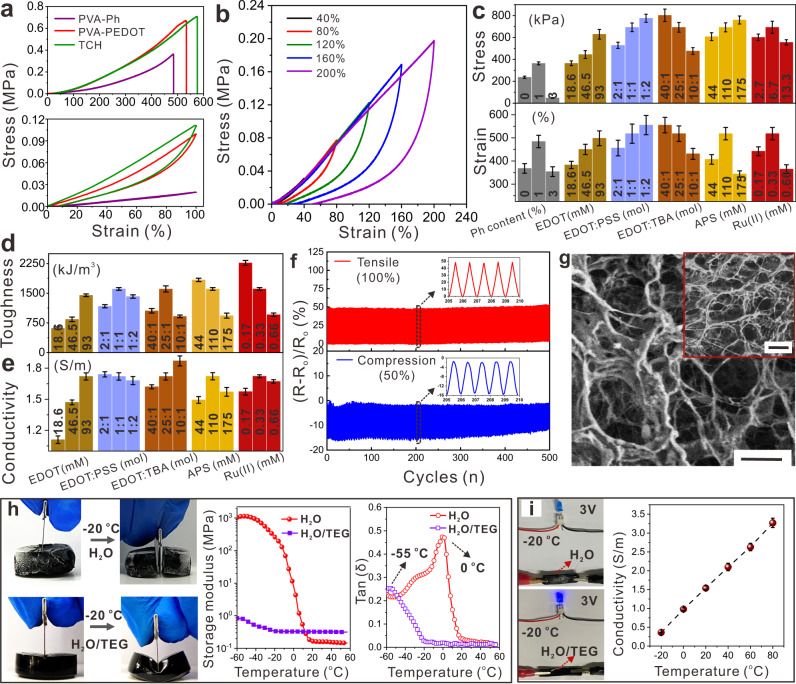
Mechanical properties and conductivity tests of TCHs. **a** Tensile tests of PVA-Ph, PVA-PEDOT (without TBA), and as-prepared TCH hydrogels. Stretching samples to maximum strains (top), strains of 100%, and then back to 0% (bottom). **b** Cyclic tensile tests of TCH to strains from 40 to 200%. The effect of preparation conditions on **c** mechanical property, **d** toughness, and **e** conductivity, respectively. **f** Resistance changes of TCHs in 500 cycles of stretching (100% strain) and compressing (50% strain) processes. **g** SEM image of freezing-dried TCHs after the cyclic tensile test in Fig. **f**. The inset image shows the sample before testing. Scale bars are 500 µm. **h** Mechanical (dynamic mechanical analysis) and toughness tests of TCHs and the hydrogel without TEG at −22 °C. **i** Optical images of powering LEDs at −22 °C by using hydrogels in **h**, and the conductivity variation of TCHs under different temperatures. TCHs were basically prepared at the following condition: [Ru(II)] = 0.33 mM, [APS] = 110 mM, [PVA-Ph] = 10 wt% with the phenol content of 1%, [EDOT] = 93 mM, [TBA] = 3.7 mM, [PSS] = 2 wt%, and TEG/H_2_O = 2/3. When evaluating the effect of one component on fabricating TCHs, other components were kept constant. Three samples were tested for each case in (**c**) and (**d**).

The effects of the preparation conditions on the mechanical properties, toughness, and conductivity were investigated by performing tensile, compression, and electrical tests. These properties are essential for advanced applications of TCHs. Figure 3a shows that although the three types of hydrogel have similar maximum stretching strains, the TCH mechanical strength and toughness are better than those of PVA-Ph and PVA-PEDOT (without TBA) hydrogels. Under cyclical extension to different strains, the TCHs showed toughening behavior typical of multinetwork structures, which is caused by the breakage of the rigid networks (Fig. 3b)^44,45^. As shown in Fig. 2a, PVA modification with phenol residues is necessary for hydrogel preparation. However, Fig. 3c and Supplementary Fig. 12 show that the mechanical properties of the TCHs initially improved and then deteriorated with increasing phenol content from 0 to 3%. That is because the high content of phenol residues increases the crosslinking density of polymeric networks, limiting the mobility of PVA chains in hydrogels to generate PVA crystallites. Also, we can observe a noticeable decrease in the stretchability of TCHs. Increasing the content of EDOT and its molar ratio with respect to PSS (but TBA) can enhance the mechanical properties and toughness by the formation of rigid PEDOT in the hydrogels (Fig. 3c, d and Supplementary Fig. 13). Although the addition of more APS slightly improved the tensile stress, the maximum strains and toughness of the TCHs decreased. This trend was also observed in tests on hydrogels with various concentrations of Ru(II) (Fig. 3c, d and Supplementary Fig. 14). A possible reason for these results is the generation of shorter PEDOT chains when the contents of APS and Ru(II) are increased (Supplementary Fig. 15).

The relationship between conductivity and the specific composition of a TCH depends on the conditions for photopolymerization of EDOT with Ru(II)/APS (Fig. 3e). We found that there were no observable changes in conductivity with changes in the concentration of Ru(II) and the molar ratio of EDOT to PSS in the hydrogel precursors. However, the conductivity varied significantly, from 0.9 to 2.0 S m^−1^, with changes in the concentrations of APS, EDOT, and TBA. The reasons for this are (i) more APS helps generate more of the oxidizing agent Ru(III), which triggers EDOT polymerization (shown in Fig. 1a), and (ii) larger amounts of EDOT and TBA increase the amount of conductive PEDOT in the TCHs. Moreover, we found that the conductivity of such hydrogels can be further improved to 50 S/m by replacing the solvent of TEG/H_2_O with pure water. These results indicate that the optimum conditions for TCH fabrication are [Ru(II)] = 0.33 mM, [APS] = 110 mM, [PVA-Ph] = 10 wt% with the phenol content of 1%, [EDOT] = 93 mM, [TBA] = 3.7 mM, [PSS] = 2 wt%, and TEG/H_2_O = 2/3. The TCHs prepared under these optimum conditions were conductive and had good fatigue resistance. Figure 3f and Supplementary Fig. 16 show that (*R* − *R*_0_)/*R*_0_ and the stress remained constant during 500 cycles of repeated stretching (100%) and compressing (50%) tests, where *R* and *R*_0_ are the corresponding resistances of the samples in the stretched (compressed) and relaxed states, respectively. These excellent mechanical and conductive properties are attributed to the stable porous networks and uniform distributions of PEDOT in TCHs. This conclusion is supported by scanning electron microscopy (SEM) examination of freeze-dried samples before and after stretching. As shown in Fig. 3g and Supplementary Fig. 17, no great difference was observed between the hydrogel networks in these two samples. When further stretching TCH to a strain of 500% and then fully releasing strain, the sample cannot recover to its original state at room temperature (Supplementary Fig. 18a, b). The reason for this is that most rigid PVA chains are stretched and fixed in TCHs. However, the stretched TCH can recover to its original length when heating it to 80 °C for 30 min. At that temperature, PVA chains are relaxed, and the size of TCH is recovered (Supplementary Fig. 18c).

Surprisingly, we found that the use of TEG/H_2_O as the solvent endowed the hydrogels with excellent anti-freezing properties because of the formation of a variety of complex supramolecular structures between TEG and H_2_O molecules in the TCHs (Fig. 3h and Supplementary Fig. 19)^22,46–48^. These structures can greatly disrupt the hydrogen bonds between H_2_O molecules, and the saturated vapor pressure of water is then significantly reduced. TEG/H_2_O does not crystallize at such low temperatures, and the freezing point decreased to −36 °C (Supplementary Fig. 20). As shown in Fig. 3h, the storage moduli (*G*′) of TCH hydrogel in TEG/H_2_O only increased by three times when lowering the temperature from 40 to −50 °C, while the *G*′ of the sample in pure water increased by about ten thousand times during the same process. By plotting the tan *δ* against temperatures, it was found that the hydrogel with H_2_O was frozen at nearly 0 °C, but the TCH was still flexible at <−20 °C. Consequently, the as-prepared hydrogel was tough, stretchable, and compressible even at temperatures −22 °C, and was not cut off by a sharp blade (Fig. 3h and Supplementary Figs. 21 and 22). The TCH was conductive and could power a light-emitting diode (LED) light at this temperature. However, the frozen hydrogels that were prepared in pure water were brittle and non-conductive at such low temperatures (Fig. 3h, i). Furthermore, TEG addition enabled flexible tuning of the TCH conductivity between 0.5 and 3.5 S m^−1^ across a wide range of environmental temperatures (−20 to 80 °C). This is useful in the design of smart and soft electronics.

### Printing 3D TCHs

**Fig. 4:**
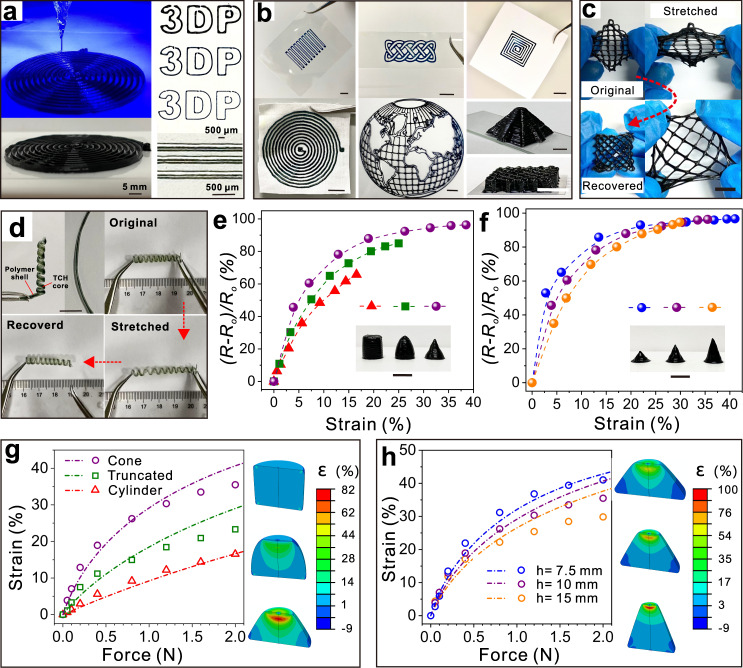
Fabricating 3D TCHs with the extruding printing technique. **a** Digital images of printed spiral TCHs via a typical extrusion 3D-printing technique (nozzle size: 22 G, moving rate: 5 mm s^−1^). **b** Digital images of different TCH patterns and 3D structures on polyethylene terephthalate, indium-tin-oxide-coated glass, paper, and cotton fabric substrates. **c** Omnidirectionally stretching a TCH mesh. The mesh was first made by extrusion 3D-printing technique with five layers on a PET substrate. Then, it was carefully peeled from the substrate, stretched, and released to its original states. **d** Making free-standing helical TCHs using co-axial 3D-printing technique with a stiff polymer. **e**, **f** Resistance changes of TCHs under different strains with different geometry shapes. **g, h** Finite element simulation of force distribution and strains in corresponding TCHs in (**e**) and (**f**). Dash lines and dots are simulated, and experimental results of compressing TCHs under different external forces applied, respectively. Inset images show the strain distributions of TCHs with the external forces applied of 0.5 and 1 N, respectively. Scale bars in (**b**), (**c**), (**e**), and (**f**) are 1 cm.

The controllable OPAP strategy is a typical photochemical process and is therefore compatible with a 3D extruding printing technique enabled 3D printing of various structured hydrogels (Supplementary Table 2). The viscous liquid precursor was put in a black syringe, on-demand extruded and simultaneously solidified by blue-light irradiation on nozzles (Fig. 4a and Supplementary Fig. 23). The extruded materials were layer-by-layer stacked to give pre-designed 3D TCHs. This printing process is template free, and pattern resolutions can be easily tuned to as low as 100 µm by selecting nozzles of different sizes or adjusting the parameters of pre-designed models. As shown in Fig. 4b, simple folded, knotted, spiral lines and a complicated world map were successfully printed on various substrates, namely smooth polyethylene terephthalate, conductive indium-tin-oxide-coated glass, rough paper, and cotton fabric. TCHs with 3D pyramidal shapes and stacked mesh structures were also made by carefully controlling the pre-designed sketches and printing parameters on target substrates. The TCH mesh, which has excellent mechanical properties and high toughness, can be peeled from the substrate, stretched, and then recover to its original state (Fig. 4c). Furthermore, TCHs can be engineered to give free-standing helical lines by co-axial 3D extrusion printing with guest polymers in the outer channel (Fig. 4d and Supplementary Fig. 24). On extrusion from a co-axial nozzle and exposure to visible light, TCHs formed in the inner channel, and transparent polymers in the outer channel, harden in the air via rapid solvent evaporation. Consequently, a stiff polymer layer is formed and can encapsulate and hold soft TCHs without any supporting components. This bilayered structure is like a spring; it can be stretched to a strain of ~130% and rapidly return to its original state (Fig. 4d).

The structures of elastic materials have significant effects on their deformation, and this leads to differences in responsiveness. Precisely tuning and optimizing their geometrical shapes via a template-free 3D-printing method can, therefore, improve the properties and functions of TCH-based devices (Supplementary Fig. 25a,b). As shown in Fig. 4e, f, the sensitivity of resistance to compression strains of the cone-like TCH with the lowest height was better than those of other 3D cylindrical, truncated, and tall-cone samples. The responsive mechanism was further investigated by using finite element analysis to simulate the strain distribution in different 3D TCHs (Fig. 4g, h and Supplementary Fig. 25c). Figure 4e, g reveals that under the same loading force, the maximum strain concentration in cone-like TCHs was about 2–30 times than those in the other two samples at the external force applied of 0.5 N. This led to the generation of larger strains and more noticeable changes in the resistance sensitivity. The lowest cone-liked structures are favorable for constructing TCH with the highest sensitivities, which can be used to detect cargo loads and recognize their shapes by comparing the changes in resistance in each sensor unit (Fig. 4f, h and Supplementary Fig. 26). These results show that 3D-printed TCHs have potential applications in high-performance flexible sensors and electronics.

### Applications of 3D TCHs in electronics

**Fig. 5:**
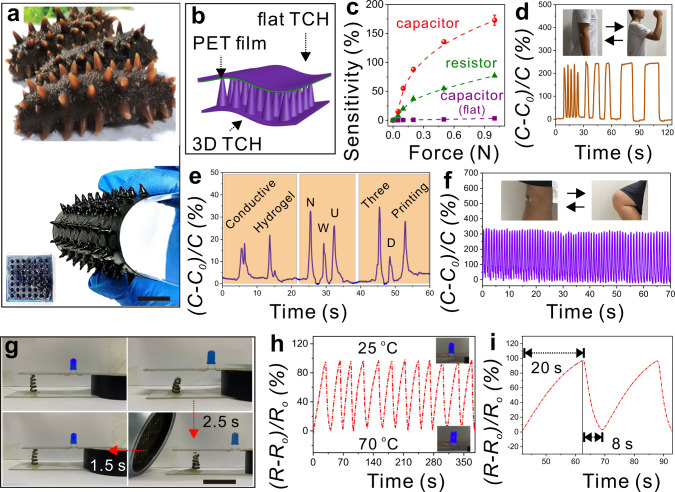
Assembling TCHs to flexible devices. **a** Bio-inspired designing a resistance sensor by 3D-printing TCH array and **b** assembling them to a 3D TCH capacitor. **c** The sensitivity of flat and as-prepared 3D TCH capacitors, and resistance sensors with different external forces. Three samples were tested for each case. The 3D TCH capacitor sensor detects more slight human motions of **d** flexing wrists with different speeds, **e** speaking, and **f** repeatedly flexing wrists and knees. **g** A temperature-responsive smart device by using the shape-memory TCH as a conductor wine. **h** The real-time variation of current and light density with alternatively heating/cooling of the TCH conductor in (**g**). **i** The time vs. resistance change in the as-prepared TCH conductor. Scale bars in (**a**) and (**g**) are 1 cm.

As a proof-of-concept, we designed a bio-inspired “sea-cucumber”-like TCH sensor by using OPAP and 3D-printing techniques. As shown in Fig. 5a, a key feature of the sea-cucumber structure is multiple hydrogel tentacles anchored to their bodies; these tentacles can detect external objects and stimuli. To mimic this hierarchical structure, a 3D TCH array with 7 × 7 cone-like units was printed and assembled to give a flexible capacitor sensor, which responded to external pressure (Fig. 5b). It is worth noting that the geometry of the cone-like TCH in this device is the same as that of the optimized structure in Fig. 4h. Considering specific applications in this study, the radius and height of cone-liked TCHs were reduced to half their original sizes. Figure 5c and Supplementary Fig. 27 show that the sensitivity to external forces of the sensor with 3D-printed TCHs was better than those of resistance and flat-capacitor sensors. This flexible device was therefore able to clearly detect slight human motions such as flexing wrists, speaking, and running (Fig. 5d–f). Importantly, previous studies showed that spatially controlling the movement and conductivity of 3D TCHs assists the fabrication of advanced 4D-printed smart devices^32,33^. This was achieved by co-axial extrusion of a typical shape-memory polymer, i.e., polylactic acid, with TCH precursors to obtain TCHs with free-standing helical structures (Supplementary Fig. 24). As shown in Fig. 5g, one end of the as-prepared helical TCH was fixed on a conductive substrate, and the other end was linked to a conductive wire. This gave a temperature-responsive actuator, which highly depended on the shape-memory properties of polylactic acid and TCH conductivity at different temperatures. When the TCH was heated, the fixed polylactic acid recovered its original shape. This drove the 3D conductive structure close to and in contact with another conductive substrate and powered a LED light (Fig. 5g and Supplementary Movie 2). Because of the fast responsivity of the polylactic acid used in the helical TCH tubes, the whole shape-memory process was completed in 4 s. More interestingly, Fig. 5h, i demonstrated that the LED intensity decreased 2.3-fold in 20 s when the heat source was removed, but it recovered on the heating again in 8 s because of the clear changes in TCH conductivity at different temperatures (Fig. 3i). Several cycles of this process can be performed by heating/cooling the TCH actuator.

## Discussion

In summary, we report the first example of a one-pot Ru(II)/APS-catalyzed OPAP for fabricating TCHs. This strategy is readily compatible with extrusion 3D-printing techniques for producing complicated structures on arbitrary substrates. These shaped hydrogels are conductive, tough, and have anti-freezing properties. They have applications in environmental pressure sensors and temperature actuators. Compared with other approaches, this OPAP method has the following advantages. First, this is an orthogonal chemistry method for designing tough hydrogels; it is based on the photopolymerization of EDOT and the phenol-coupling reaction of tyramine-modified PVA. Natural polymers (e.g., gelatin, silk protein, and bovine serum albumin) and other phenol-modified polymers can therefore be used for fabricating tough hydrogels. Some functional polymers can be introduced into this system to design, for example, shape-memory devices and actuators. Second, the use of visible-light irradiation and biocompatible PVA makes it possible to pattern or print cells, proteins, and enzyme-containing inks with desired structures at room temperature. This enables advanced applications in the fields of artificial organs and tissue engineering. Third, the whole fabrication process is achieved under short light irradiation and can therefore be used to make shaped hydrogels by combining this process with typical well-known lithography and printing techniques. These include, but are not limited to, those used in this work. Fourth, because of their inherent electrochromic and electrochemical features, these PEDOT-containing tough hydrogels can be used as display or energy-storage components of smart devices. Because of their excellent anti-freezing properties, these devices operate well even at low temperatures. We believe that this OPAP strategy opens up new horizons in research on multinetwork tough materials and will inspire the simple design of conductive hydrogels with high-resolution patterns and complicated structures. The OPAP method and synthesized TCHs have many potential applications in the biological and materials sciences.

## Methods

3,4-Ethylenedioxythiophene, polyvinyl alcohol (Mw ~145,000 g mol^−1^, Aladdin), tris(bipyridine)ruthenium(II) chloride (ACROS), poly(styrenesulfonic acid sodium salt) (Mw ~70,000 g mol^−1^), PEDOT:PSS (Clevios^TM^ PH1000, Heraeus Electronic Materials) and thiopheneboronic acid (Sigma-Aldrich), polylactic acid (4032D, NatureWorks), and other chemicals (Sinopharm Chemical Reagent Co. Ltd.) were purchased and used without further purification.

### Synthesis of tyramine-modified PVA (PVA-Ph)

A typical synthesis process of PVA-1% Ph (i.e., PVA with 1% phenol in molecular structure) is as follows: 10 g (0.7 mmol) of PVA powder was first mixed with 100 ml of N-methylpyrrolidone at 100 °C by vigorously mechanical stirring. When PVA was fully dissolved, the temperature was decreased to 70 °C. 4-Dimethy-laminopyridine (0.28 g, 2.3 mmol) and succinic anhydride (0.24 g, 2.4 mmol) were then added to the above mixture for 24 h stirring. After being cooled down to room temperature, the achieved mixture was added dropwise to ethyl acetate (~500 ml). The precipitate was collected and dried at 70 °C for 2 h in an oven. The obtained product was fully re-dissolved in 100 ml of DI water at 70 °C by mechanical stirring for 2 h. When decreasing the temperature to 30 °C, N-hydroxysuccinimide (0.56 g, 4.8 mmol), tyramine (0.66 g, 4.8 mmol), and 1-ethyl-3-(3-dimethylaminopropyl)carbodiimide (0.92 g, 4.8 mmol) were added to the above solution. After further 24 h stirring, the resultant mixture was introduced dropwise to ethanol (~500 ml). The generated precipitate was collected and washed by ethanol twice. The final PVA-1% Ph was obtained by completely drying the collected precipitate at 70 °C in an oven.

### Fabrication of TCHs

In a typical fabricating process, 1 g of PVA-*x*Ph (*x* stands for the percentage of phenol in the molecular structure of PVA) was fully dissolved in 10 ml mixed solution of DI water and TEG via rapidly stirring at 70 °C. Different amounts of PSS, EDOT, and TBA (dissolved in 200 ul DMSO) were then added, following by magnetically stirred for 30 min at 70 °C until a homogenous solution was achieved. After being cooled to room temperature, the desired amount (~330 mg, 0.2 mmol) of p-toluenesulfonic acid was dissolved in the solution for tuning the PH value to 1–2. The catalyst of Ru (II) and APS were then added according to a predetermined concentration. The hydrogel precursor was finally obtained after completely dissolving the catalyst via magnetic stirring for ~5 min at room temperature. It should be noted that the whole process for the preparation of the precursor must be proceeded in a sealed container to prevent water evaporation. After degassing in a centrifuge at a rotating speed of 13,000 rpm, the precursor was cast into a Teflon mold to form TCHs by exposing to visible light.

3D printing of TCHs was basically conducted on a custom-designed 3D printer with a nozzle of 390 μm (22 G), printing speed of 5 mm/min, applied pressure around 0.4 MPa. All 3D structures are designed by Sketchup software, and the corresponding fabrication conditions are listed in Supplementary Table 2. During the 3D-printing process, the prefabricated precursor was first mixed with a little amount (3 wt%) of hydroxyethylcellulose (Q10) to acquire suitable printing viscosity. The precursor was then put into a 10-ml syringe with nozzles of the desired diameter after degassing by the centrifuge at 13,000 rpm. Next, the syringe was connected to the pressure supply and mounted on the head of the robot. User-defined TCH-based structures could be obtained by accurate control of the applied pressure, moving direction, and velocity of the robot with visible light irradiation. When fabricating bilayer-structured TCHs, a co-axial nozzle (16G/12G) was used, and a dichloromethane solution of polylactic acid in the outer channel was co-extruded with TCH precursors and exposed them to visible light as same as that of fabricating above 3D structures. The detailed printing parameters of each structure are listed in Supplementary Table 2.

### Characterizations

In situ FT-IR (Bruker, INVENIO R) was carried out to monitor the real-time polymerization of EDOT under visible light (452 nm) by inserting the probe into the precursor for 15 min. The scan was performed every 3 s, and the resolution was set as 4 cm^−1^. Ultraviolet spectra were characterized by an ultraviolet spectrophotometer (PRESEE, TU-1810) at a scan range of 50 nm min^−1^. Differential scanning calorimetry (TA Instruments) was employed to characterize the freezing point of TCHs under a nitrogen atmosphere (50 mL min^−1^) in a temperature range of 25–70 °C with a rate of 5 °C min^−1^. Dynamic mechanical analysis (Netzsch) was performed to evaluate the thermal–mechanical properties of hydrogels at a frequency of 1 Hz and a temperature range of −70~60 °C with a rate of 3 °C min^−1^. X-ray diffraction (D8 Advance) was employed to characterize the formation of PVA crystallites in TCHs during the freezing process. SEM was used to observe the morphologies of samples. TCHs were freeze-dried after immersing in DI water for 24 h to remove TEG. The surface of the samples was then sputter-coated with gold before observation. The gelation process of the hydrogel precursor under blue light was evaluated by a rotational rheometer (Anton Paar MCR302) assembled with optical modules and a 20-mm diameter steel parallel-plate geometry. The testing was conducted at room temperature with a strain of 1% at 10 Hz. All rheological characterizations were performed after 1 min of equilibration. The conductivity of TCHs (10 mm × 50 mm × 1 mm) was measured by a two-probe testing assembly with a digital multimeter (Keithley, 6517B). That was calculated from the formula of *σ* = *L*/*RS* (*σ* is the conductivity, *L* and *S* are the lengths and the area of cross-section, respectively). Five samples were tested for each composition. To measure its conductivity at different temperatures, the sample is tightly linked with two copper tapes and sealed by parafilm to prevent water evaporation from hydrogels. After that, the sample is put into a temperature-controlled box, and the conductivity is recorded when the sample is put at the corresponding temperature for 10 min. Capacitance was measured by the precision LCR meter (TH2829B) with an applied voltage of 1.0 V (1 kHz). The mechanical tests of TCHs (10 mm × 50 mm × 1 mm) were performed on a tensile clamp (MTS, Insight 50) at a speed of 20 mm min^−1^ at room temperature. Toughness was calculated from the integration of the strain-stress curves in tensile tests.

Further details on the methods are available in the Supplementary Information.

## Supplementary information

Supplementary Information

Description of Additional Supplementary Files

Supplementary Movie 1

Supplementary Movie 2

## Data Availability

All the data supporting the findings in this study are available in the paper and Supplementary information files. All the data related to this paper are available from the corresponding authors upon reasonable request.
