# Supplementary material for: Orthogonal photochemistry-assisted printing of 3D tough and stretchable conductive hydrogels
Source: Nat Commun. 2021 Apr 7;12:2082. doi: 10.1038/s41467-021-21869-y (PMC8027177; doi:10.1038/s41467-021-21869-y)
Supplement: Supplementary file 1 — Supplementary Information [file 41467_2021_21869_MOESM1_ESM.pdf]

Supplementary Information

Orthogonal Photochemistry-assisted Printing of 3D Tough and Stretchable Conductive Hydrogels

Hongqiu Wei[1], Ming Lei[2], Ping Zhang[1], Jinsong Leng[3], Zijian Zheng[4] & You Yu[1,*]

Correspondence to: yuyou@nwu.edu.cn

Affiliations:

[1]Key Laboratory of Synthetic and Natural Functional Molecule Chemistry of the Ministry of Education, College of Chemistry and Materials Science, Northwest University, Xi'an 710069, China.

[2]School of Astronautics, Northwestern Polytechnical University, Xi'an, China.

[3]Center for Composite Materials and Structures, Department of Astronautical Science and Mechanics, Harbin Institute of Technology, Harbin, China.

[4]Nanotechnology Center, Institute of Textiles and Clothing, The Hong Kong Polytechnic University, HongKong, China.

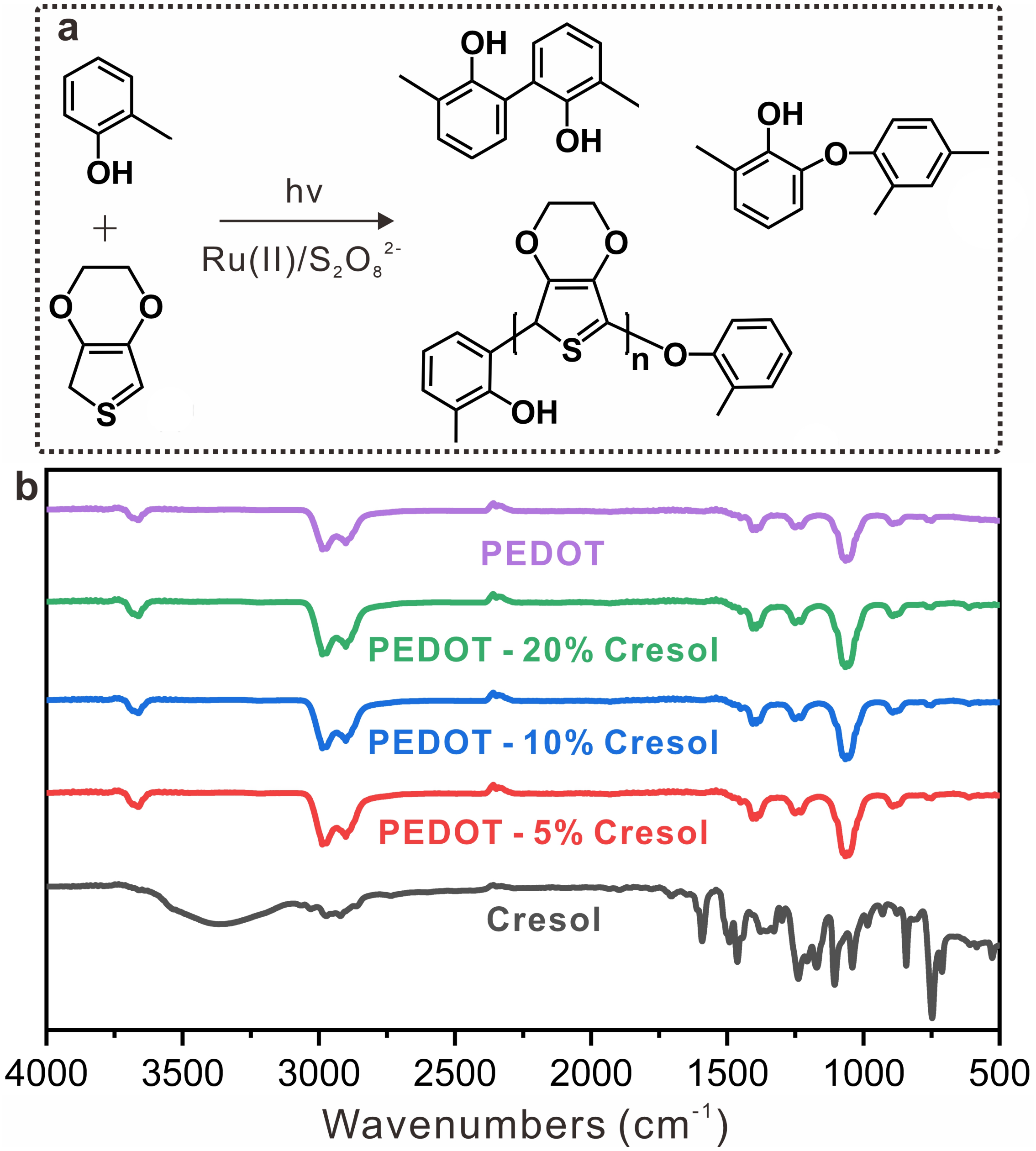

Supplementary Figure 1. The possible photoreaction during the formation of TCHs. (**a**) Possible products generated between cresol (2-hydroxytoluene) and EDOT. (**b**) FT-IR spectra of products of cresol and the mixture of EDOT and cresol. [EDOT] = 93 mM, [Ru(II)] = 0.33 mM, [APS] = 109.6 mM, volume ratio of H_2O/TEG = 3:2, and the irradiation intensity was 15 mW cm^{-2}. Cresol was mixed with EDOT in the precursor with molar ratios of 0, 5%, 10%, and 20%, respectively.

Supplementary Note 1

To further identify these orthogonal reactions, we design a control experiment to detect the possible reaction between EDOT and phenol residues. As shown in Supplementary Figure 1a, this phenol derivative supplies one reaction site to react with EDOT or themselves. Cresol (2-hydroxytoluene) is mixed with EDOT, Ru(II), APS in TEG/H_2O, and then is exposed to visible light for three minutes. After that, excessive ethanol is used to wash the products for removing the unreacted cresol and diphenol products. Finally, only the PEDOT-contained polymer is obtained. Therefore, the possible reaction between phenol and EDOT can be easily studied by using FT-IR spectroscopy. Supplementary Fig. S1b showed that no characteristic peaks of phenol derivates were observed in the FT-IR spectrum of the PEDOT-contained polymer. And there is no obvious difference in PEDOT-contained polymers with varied contents of cresol. This comparison indicates that 2-hydroxytoluene doesn't react with EDOT. Therefore, it is reasonable to conclude that, as for the OPAP strategy, PVA-Ph cannot react with EDOT. Based on this discussion, we believe this Ru(II)-based photochemistry is an orthogonal process.

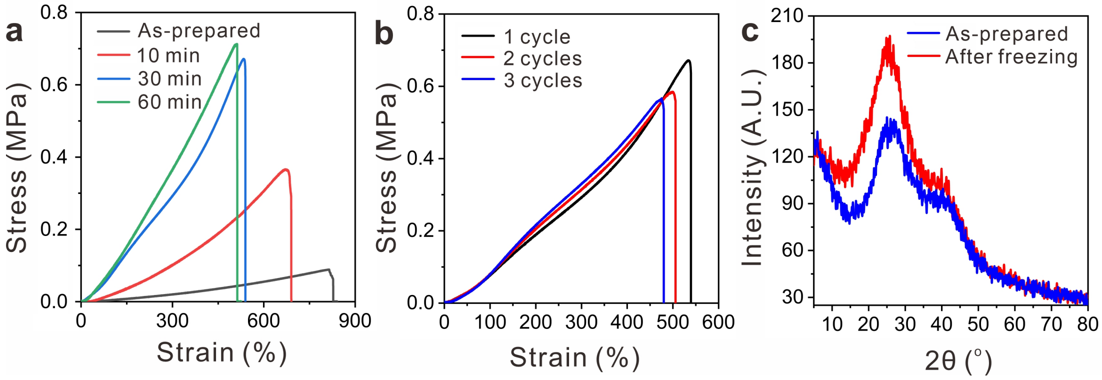

Supplementary Figure 2. Effect of freezing post-treatment on mechanical property and structure of TCHs. (**a**) Strain-stress curves with different freezing time (0 ~ 60 min). (**b**) Strain-stress curves with different freezing-thawing cycles (30 min). Before testing, samples were first frozen at -22 °C for 30 min and then thawed at room temperature for two hours. After that, this procedure was repeated two or three cycles. (**c**) The X-ray diffraction characterization of hydrogels before and after freezing-thawing post-treatment. The characteristic peaks at 25° and 40° belong to the crystallized PVA in TCHs. Compared with the as-prepared sample, the intensity of the XRD peaks of freezing-treated TCH increases, indicating more PVA crystallites generated in hydrogels at low temperatures. Three samples were tested for each case in (a) and (b).

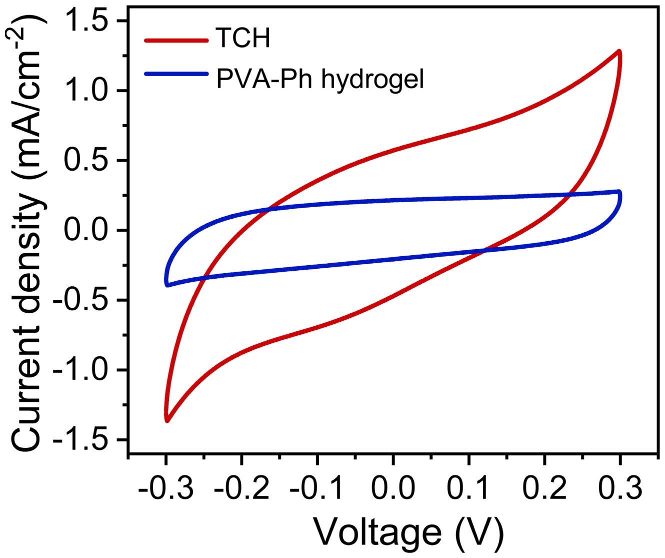

Supplementary Figure 3. Cyclic voltammetry (CV) characterization of TCH and PVA-Ph hydrogel. The CV testing was performed by using a potentiostat/galvanostat electrochemical instrument with a scan rate of 20 mV s^{-1}. The saturated calomel electrode was used as a reference electrode. The mixed solution of H_2O and TEG (volume ratio is 3/2) with 1.0 M TsOH was employed as the supporting electrolyte. The components PVA-Ph hydrogel includes [PVA-1%Ph] = 10 wt%, [Ru(II)] = 0.33 mM, [APS] = 109.6 mM and volume ratio of H_2O/TEG = 3:2. The testing TCH was prepared at [PVA-1%Ph] = 10 wt%, [EDOT] = 93 mM, [PSS] = 2 wt%, [TBA] = 3.7 mM, [Ru(II)] = 0.33 mM, [APS] = 109.6 mM and volume ratio of H_2O/TEG = 3:2.

Supplementary Note 2

As a typical electroactive polymer, PEDOT has been extensively used for fabricating electrochemical sensors, pseudocapacitors, polymer solar cells, and so on. In this experiment, PEDOT in TCH is reversibly oxidized or reduced during charge/discharge processes, which supplies more space for loading/releasing cations compared with the PVA-Ph hydrogel. This conclusion is strongly supported by the CV characterization of TCH and PVA-Ph hydrogels. As shown in Supplementary Fig. 3, the absolutely integral areas of CV curves can be used to evaluate their electrochemical properties. By comparing the integral areas of these two curves, the pseudocapacitance of TCH is 2.5 times that of PVA-Ph hydrogel, when ~1.5 wt.% of PEDOT is introduced into the sample. The CV of TCHs further shows broad and stable anodic and cathodic peaks under varying potentials, suggesting non-diffusional redox processes of TCHs[1, 2]. These achieved results indicate that the introduction of PEDOT can improve the electrochemical property of as-prepared hydrogels. Moreover, we can reasonably conclude that this property will be further enhanced by introducing more electroactive PEDOT in TCHs.

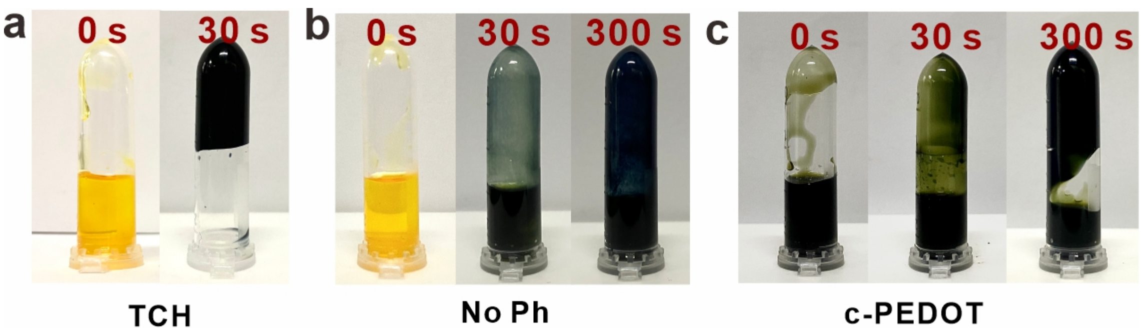

Supplementary Figure 4. Effect of Phenol content and c-PEDOT on the formation of TCHs. (**a**) TCH precursor ([PVA-1%Ph] = 10 wt%, [EDOT] = 93 mM, [PSS] = 2 wt%, [TBA] = 3.7 mM, [Ru(II)] = 0.33 mM, [APS] = 109.6 mM and volume ratio of H_2O/TEG = 3:2). (**b**) Hydrogel precursor with no Ph ([PVA] = 10 wt%, [EDOT] = 93 mM, [PSS] = 2 wt%, [TBA] = 3.7 mM, [Ru(II)] = 0.33 mM, [APS] = 109.6 mM and volume ratio of H_2O/TEG = 3:2). (**c**) Commercial PEDOT-contained hydrogel precursor ([PVA-1%Ph] = 10 wt%, [PEDOT:PSS] = 1 wt%, [TBA] = 3.7 mM, [Ru(II)] = 0.33 mM, [APS] = 109.6 mM and volume ratio of H_2O/TEG = 3:2).The photographs were taken by placing the 1 ml precursor of (a), (b) and (c) under blue light irradiation with different times (0 s, 30 s, and 300 s, respectively.)

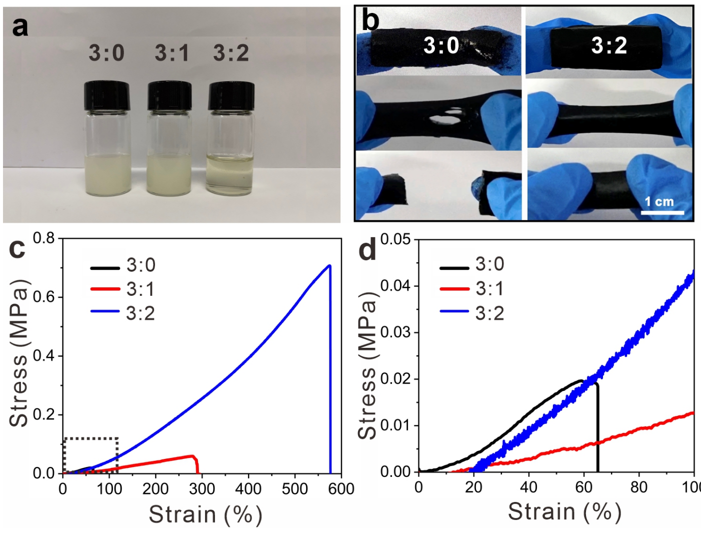

Supplementary Figure 5. Mechanical properties of TCHs prepared with different volume ratios of H_2O/TEG. (**a**) Digital images of TCH precursors prepared with different H_2O/TEG in volume (3:0, 3:1, and 3:2, respectively). (**b**) Elongation experiment of TCHs prepared with a volume ratio of H_2O/TEG = 3:0 and 3:2, respectively. (**c**) The typical strain-stress curves of TCHs prepared with different H_2O/TEG in volume (3:0, 3:1, and 3:2, respectively). (**d**) The magnification of the selected part in (c). Other ingredients in the TCHs precursor are [PVA-1%Ph] = 10 wt%, [EDOT] = 93 mM, [PSS] = 2 wt%, [TBA] = 3.7 mM, [Ru(II)] = 0.33 mM and [APS] = 109.6 mM.

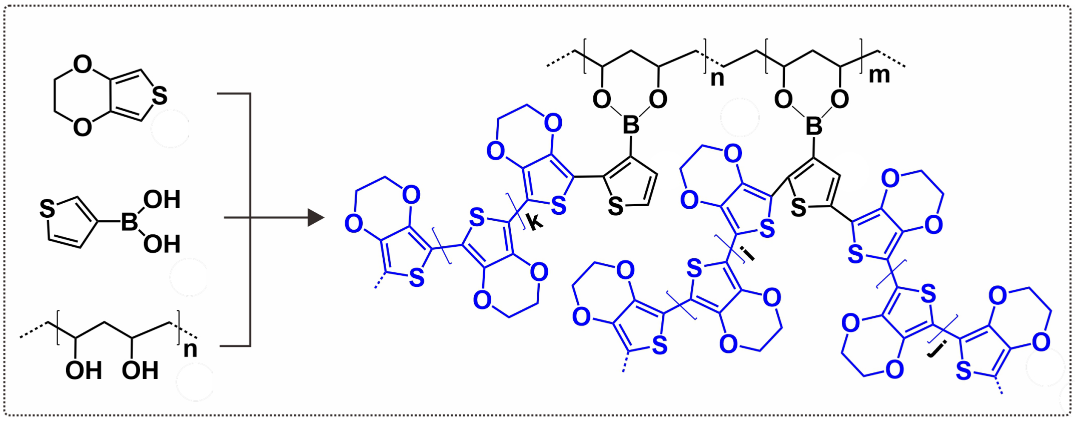

Supplementary Figure 6. Schematic illustration of grafting PEDOT onto PVA chains via boronic acid ester chemistry in TCHs.

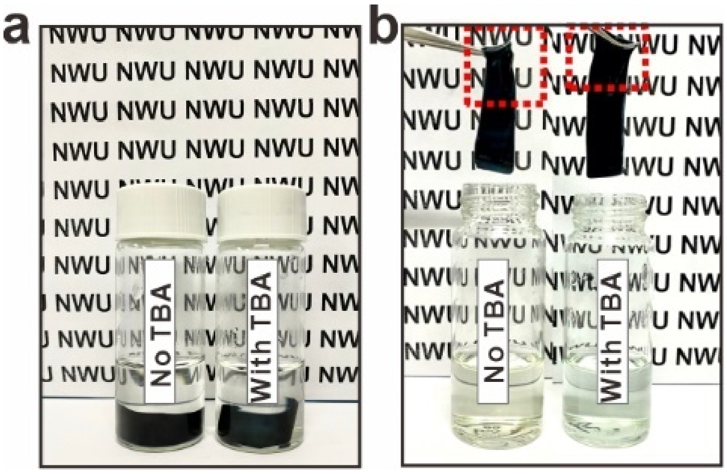

Supplementary Figure 7. Effect of TBA on the mechanical strength of TCHs in water. Samples in DI water with immersion times of (**a**) 0 and (**b**) 48 h, respectively. The digital images showed that although these two samples can be pulled from the water, the sample without TBA was soft and deformed compared with that with TBA (3.7 mM). Other components in these two hydrogels were [PVA-1%Ph] = 10 wt%, [EDOT] = 93 mM, [PSS] = 2 wt%, [TBA] = 3.7 mM, [Ru(II)] = 0.33 mM and [APS] = 109.6 mM.

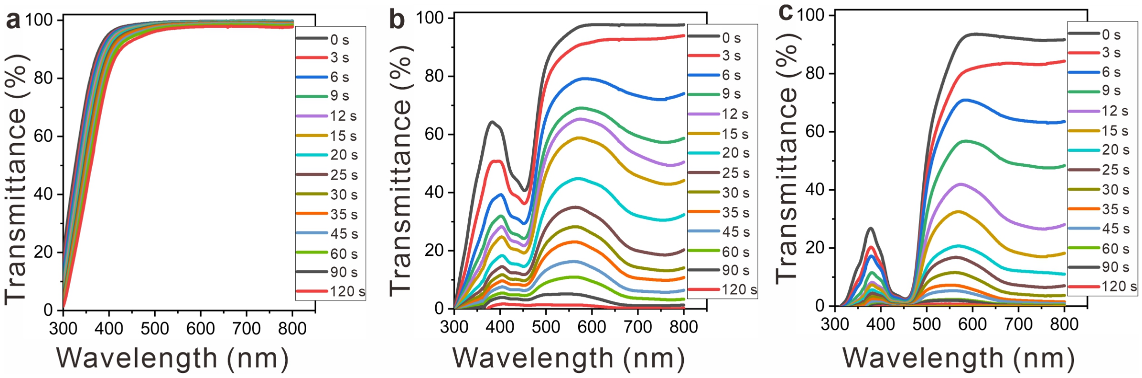

Supplementary Figure 8. UV-vis transmittance of EDOT/PSS solution with different catalysts. (**a**) [$FeCl_3$] = 2.7 mM, [APS] = 109.6 mM. (**b**) [Ru(II)] = 2.7 mM, [APS] = 109.6 mM. (**c**) [Ru(II)] = 0.33 mM, [APS] = 109.6 mM. Other components are [EDOT] = 46.5 mM, [PSS] = 1 wt%, volume ratio of H_2O/TEG = 3:2.

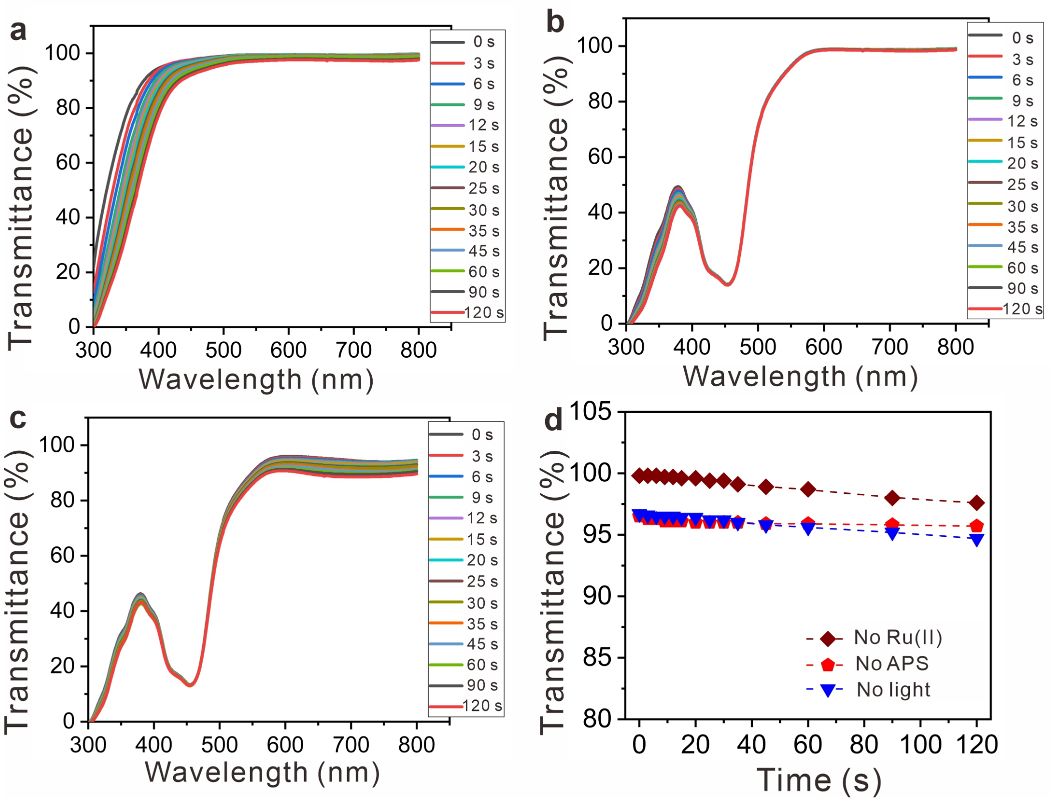

Supplementary Figure 9. UV-vis transmittance of EDOT/PSS solution under different control preparation conditions. (**a**) [Ru(II)] = 0.33 mM, No APS, light irradiation. (**b**) [APS] = 109.6 mM, No Ru(II), light irradiation. (**c**) [Ru(II)] = 0.33 mM, APS = 109.6 mM, no light irradiation. The scan was ranged from 300 nm to 800 nm, with a rate of 50 nm min^{-1}. (**d**) The transmittance of EDOT/PSS solution @ 550 nm change with reaction time under the initiating condition of (a), (b), and (c). Other components are [EDOT] = 46.5 mM, [PSS] = 1 wt%, volume ratio of H_2O/TEG = 3:2.

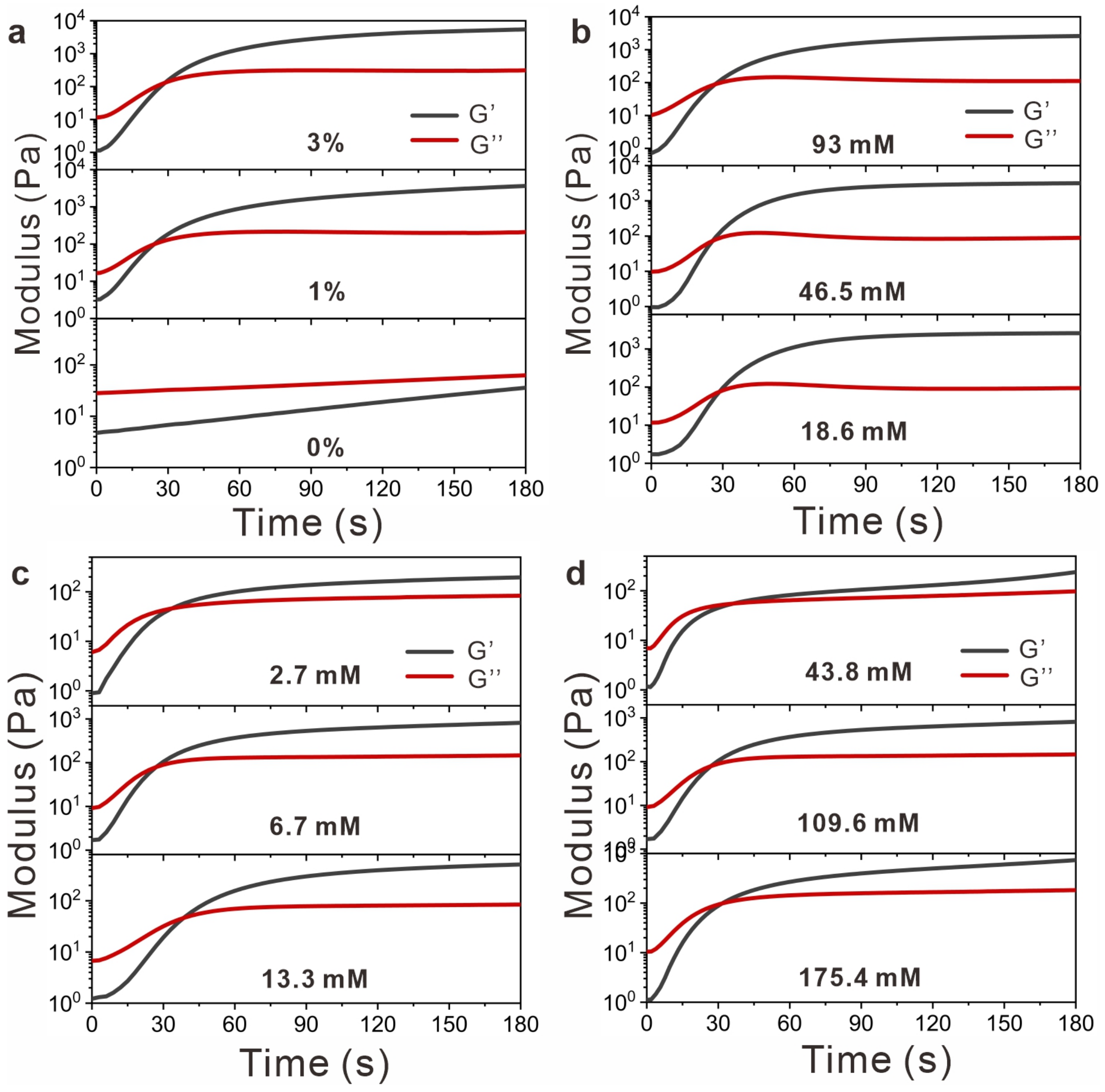

Supplementary Figure 10. Real-time rheology characterizations of TCHs hydrogels. (a) TCHs with different contents of Ph in PVA. The weight ratio of PVA-*x*Ph in TCH is 10 wt%. The content of other components are [EDOT] = 93 mM, [PSS] = 2 wt%, [TBA] = 3.7 mM, [Ru(II)] = 0.33 mM, [APS] = 109.6 mM and volume ratio of H_2O/TEG = 3:2. (**b**) TCHs with different concentrations of EDOT. Other components are [PVA-1%Ph] = 10 wt%, [PSS] = 2 wt%, [TBA] = 3.7 mM, [Ru(II)] = 0.33 mM, [APS] = 109.6 mM and volume ratio of H_2O/TEG = 3:2. (**c**) TCHs with different concentrations of Ru(II). Other components are [PVA-1%Ph] = 10 wt%, [EDOT] = 93 mM, [PSS] = 2 wt%, [TBA] = 3.7 mM, [APS] = 109.6 mM and volume ratio of H_2O/TEG = 3:2. (**d**) TCHs with different concentrations of APS. Other components are [PVA-1%Ph] = 10 wt%, [EDOT] = 93 mM, [PSS] = 2 wt%, [TBA] = 3.7 mM, [Ru(II)] = 0.33 mM and volume ratio of H_2O/TEG = 3:2.

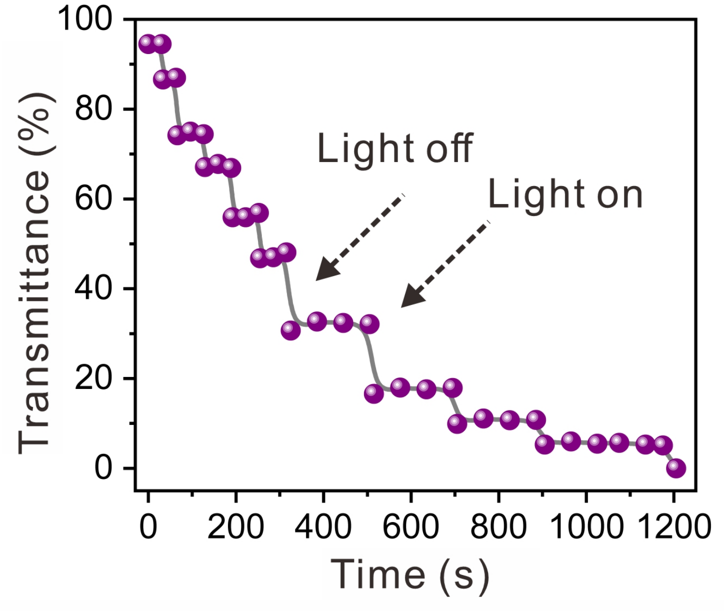

Supplementary Figure 11. The transmittance of TCH precursor @550 nm under intermittent blue light irradiation. ([PVA-1%Ph] = 10 wt%, [EDOT] = 46.5 mM, [PSS] = 1 wt%, [TBA] = 1.86 mM, [Ru(II)] = 0.33 mM, [APS] = 109.6 mM and volume ratio of H_2O/TEG = 3:2).

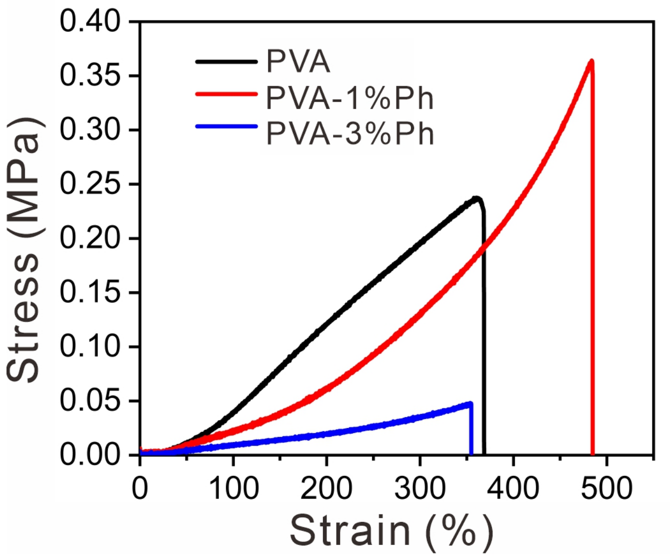

Supplementary Figure 12. Typical strain-stress curves of PVA and PVA-*x*Ph hydrogels with different Ph contents. The weight ratio of PVA-*x*Ph in TCH is 10 wt%. The content of other components are [EDOT] = 93 mM, [PSS] = 2 wt%, [TBA] = 3.7 mM, [Ru(II)] = 0.33 mM, [APS] = 109.6 mM and volume ratio of H_2O/TEG = 3:2. Three samples were tested for each case.

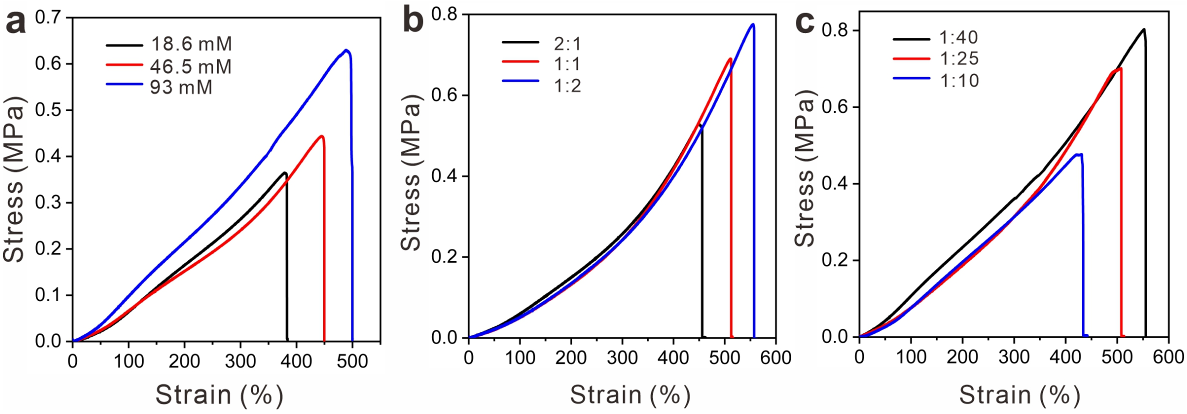

Supplementary Figure 13. Typical strain-stress curves of TCHs with different components of hydrogel precursors. (**a**) TCHs with different concentrations of EDOT. The content of other components are [PVA-1%Ph] = 10 wt%, [PSS] = 2 wt%, [TBA] = 3.7 mM, [Ru(II)] = 0.33 mM, [APS] = 109.6 mM and volume ratio of H_2O/TEG = 3:2. (**b**) TCHs with different molar ratio of EDOT: PSS ([EDOT] = 93 mM). The content of other components are [PVA-1%Ph] = 10 wt%, [TBA] = 3.7 mM, [APS] = 109.6 mM, [Ru(II)] = 0.33 mM and volume ratio of H_2O/TEG = 3:2. (**c**) TCHs with different molar ratio of EDOT: TBA ([EDOT] = 93 mM). The content of other components are [PVA-1%Ph] = 10 wt%, [PSS] = 2 wt%, [Ru(II)] = 0.33 mM, [APS] = 109.3 mM and volume ratio of H_2O/TEG = 3:2. Three samples were tested for each component.

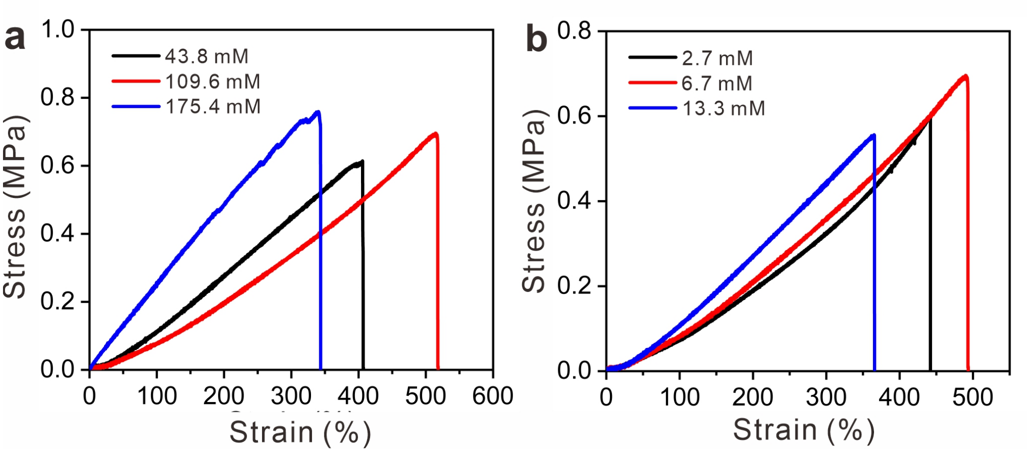

Supplementary Figure 14. Tensile tests of TCHs hydrogels with different catalysts. (**a**) TCHs with different concentrations of APS. The content of other components were [PVA-1%Ph] = 10 wt%, [EDOT] = 93 mM, [PSS] = 2 wt%, [TBA] = 3.7 mM, [Ru(II)] = 0.33 mM and volume ratio of H_2O/TEG = 3:2. (**b**) TCHs with different concentrations of Ru(II). The content of other components are [PVA-1%Ph] = 10 wt%, [EDOT] = 93 mM, [PSS] = 2 wt%, [TBA] = 3.7 mM, [APS] = 109.3 mM and volume ratio of H_2O/TEG = 3:2. Three samples were tested in each case.

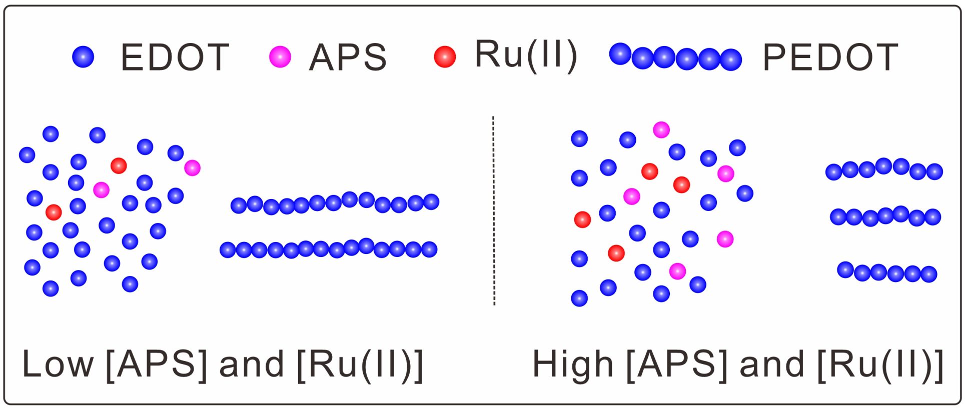

Supplementary Figure 15. Schematic diagram of the effect of concentration of APS and Ru(II) on the polymerization of EDOT. As shown in this figure, lower concentrations of [APS] and [Ru(II)] generate less Ru(III) ions to trigger the polymerization of EDOT. The resultant PEDOT has a longer chain, which helps enhance the mechanical properties of the TCH. Instead, [APS] and [Ru(II)] in higher concentrations could trigger more Ru(III) ions to initiate the polymerization of EDOT that leading to the formation of shorter PEDOT chains, which have less contribution in improving the mechanical properties of TCHs.

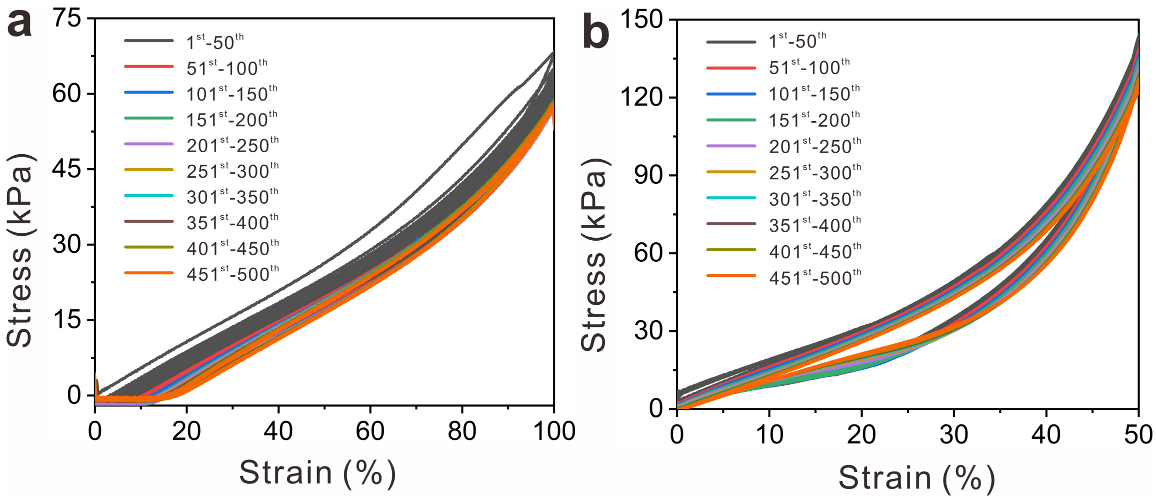

Supplementary Figure 16. Cyclic tests of mechanical properties of TCHs. (**a**) Strain-stress curves for the cyclic tensile test (100% strain). TCH sample with a dimension of 10 mm × 50 mm × 1 mm was fixed onto a tensile clamp. The testing length was kept as 30 mm. The upper clamp first stretched the sample for 30 mm, following by a return to its original position at a rate of 20 mm min^{-1}. Such a tensile process was repeated for 500 cycles. (**b**) Strain-stress curves for cyclic compression test (50% strain). A cylindrical sample with a radius of 10 mm and a height of 10 mm was placed onto a compression holder. The upper holder has first compressed the sample for 5 mm and then recovered to its initial testing position at a rate of 50 mm min^{-1}. This compression process was repeated for 500 cycles.

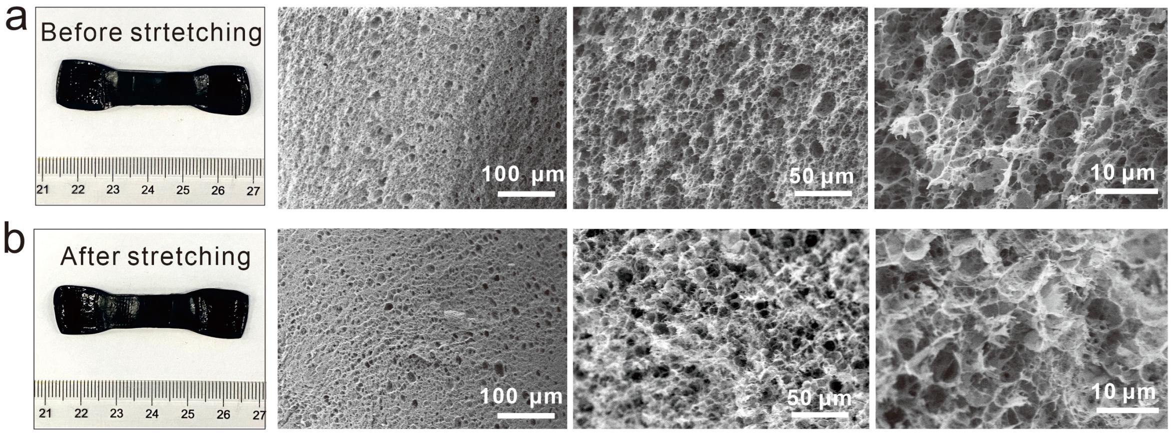

Supplementary Figure 17. Morphologies of before and stretched TCHs with a strain of 100%.

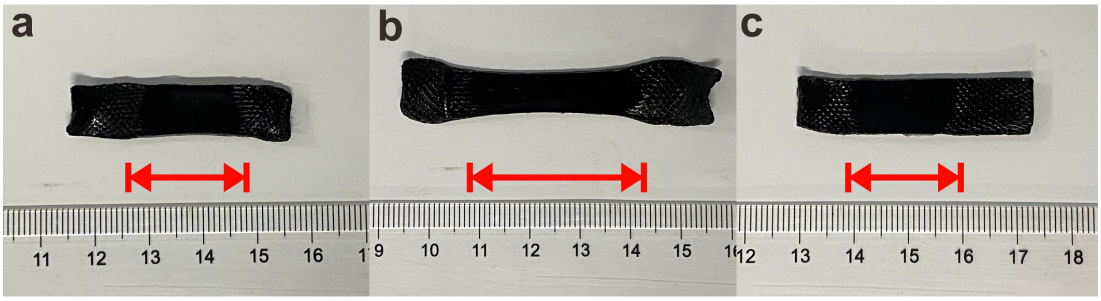

Supplementary Figure 18. Recovery Capability of TCHs when stretching to a strain of 500%. The sample was first fixed on the tensile equipment (Fig. **a**) and then stretched to a determined strain of 500%. After releasing the strain, the stretched sample in Fig. **b** was encapsulated by parafilm to prevent the water evaporation and put into an oven at 80 ºC for 30 min. Finally, the sample was taken out and cooled to room temperature in the air (Fig. **c**). From these images, we observed that TCH partially recovered to its original state due to rigid PVA chains, which were stretched and fixed at room temperature. However, the sample fully recovered to its original state with heating treatment because PVA chains in the TCH were fully relaxed at high temperatures (e.g., 80 ºC).

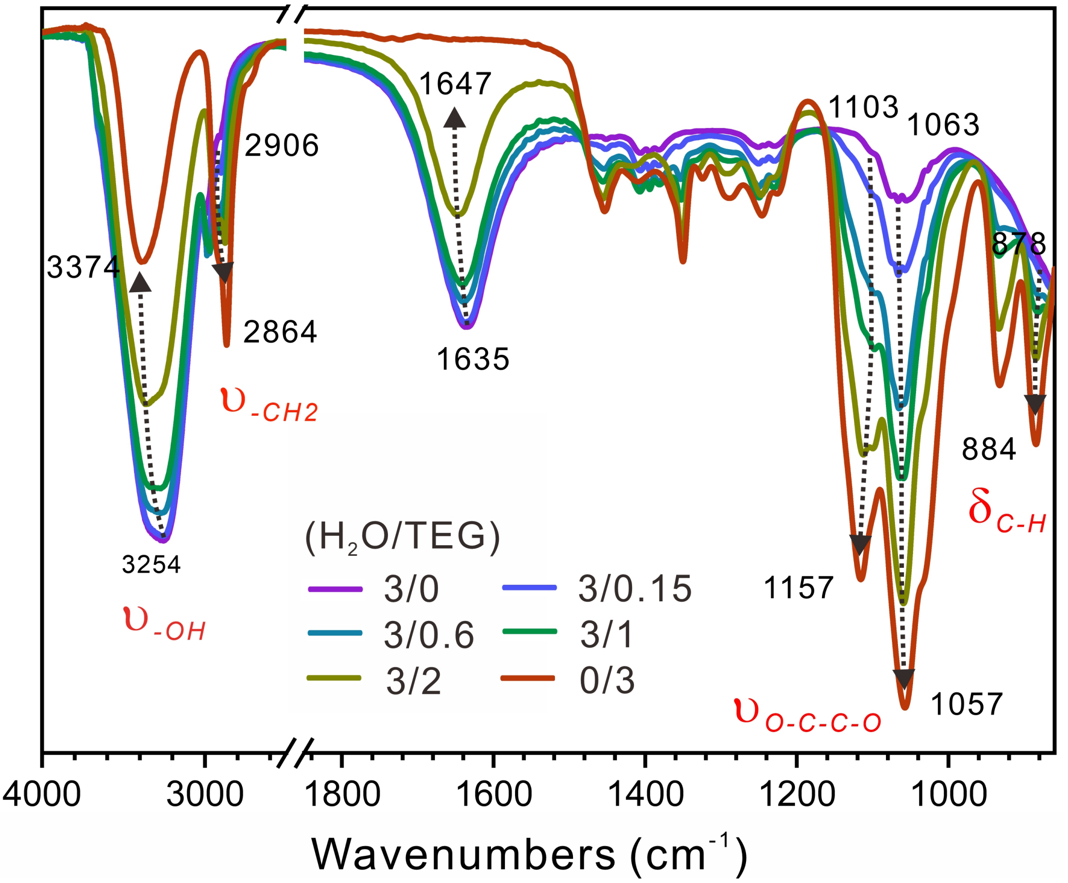

Supplementary Figure 19. ATR-FTIR characterization of forming supramolecular structures in the mixed solvents of H_2O and TEG. It was found the characteristic peaks of TEG in the ATR-FTIR spectra were shifted when adding different portions of TEG to water. This result was accordant with the previous report in a similar study[3]. In detail, the vibration of OH bonds gradually shifted from 3254 to 3374 cm^{-1}, but the vibration of -CH_2- in TEG shifted from 2906 to 2864 cm^{-1}. The vibration shift and intensity increase behaviors of O-C-C-O in TEG were also observed at the range of 1200 to 1000 cm^{-1}. Moreover, the absorption of water between 1800 to 1500 cm^{-1} gradually decreased, and the peak shifted from 1635 cm^{-1} to 1647 cm^{-1}. The reason for this can be assigned to the formation of hydrogen bonds between water and TEG, and more supramolecular structures formed when increasing the content of TEG to water.

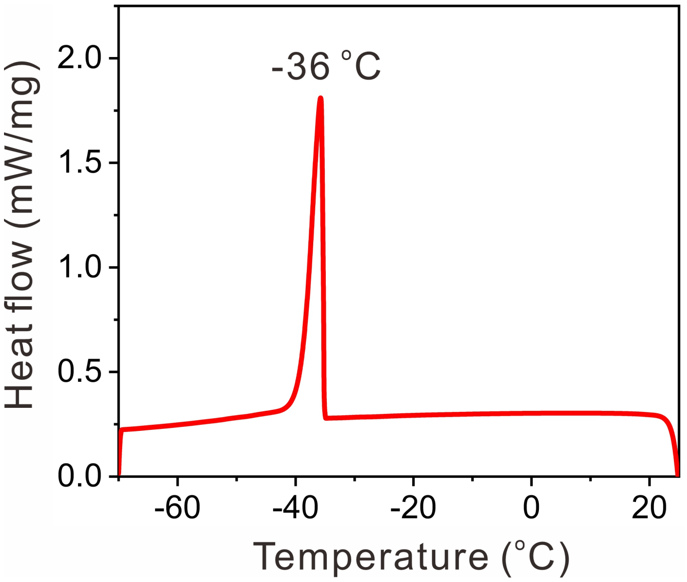

Supplementary Figure 20. The freezing-point characterization of TCHs by differential scanning calorimetry (DSC).

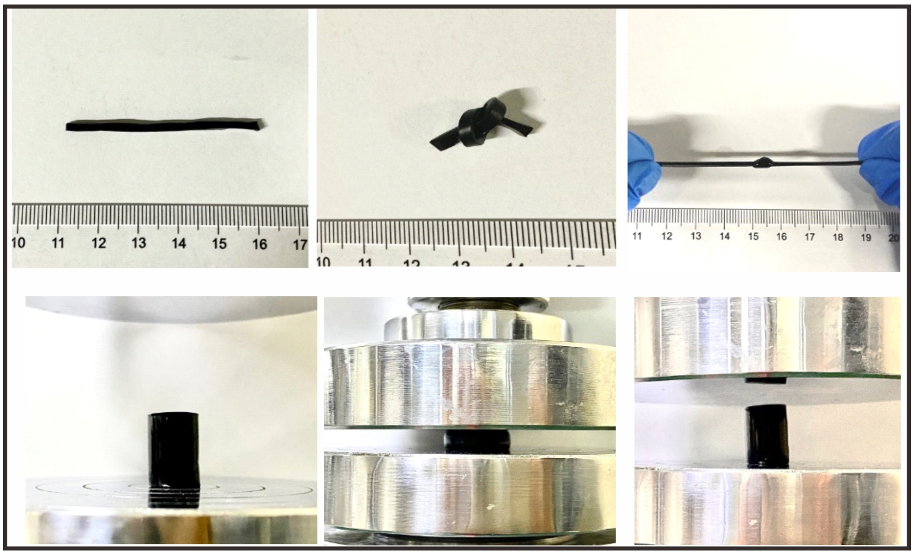

Supplementary Figure 21. Stretching, knotting, and compressing TCHs at a low temperature (~22 °C).

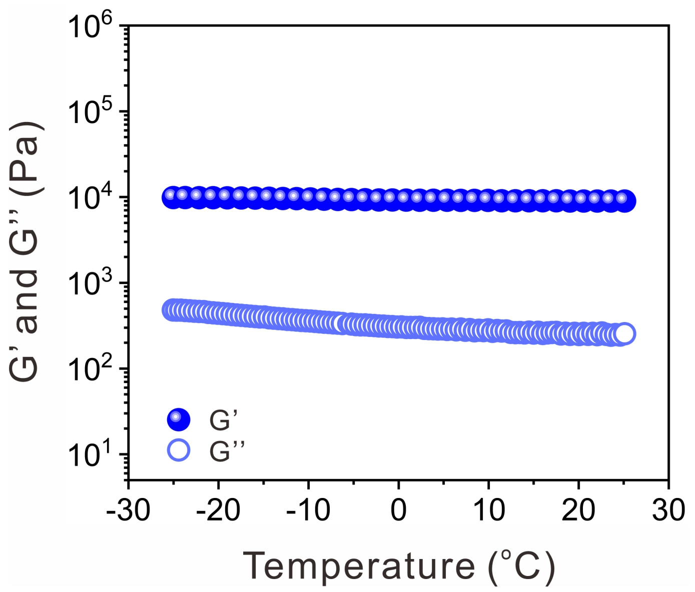

Supplementary Figure 22. Rheology characterization of TCHs with varying temperatures from -25 °C to 25 °C. There is no noticeable change in G′ and G″ when varying temperatures, and this result indicates TCHs still have good mechanical properties at low temperatures (<0 °C).

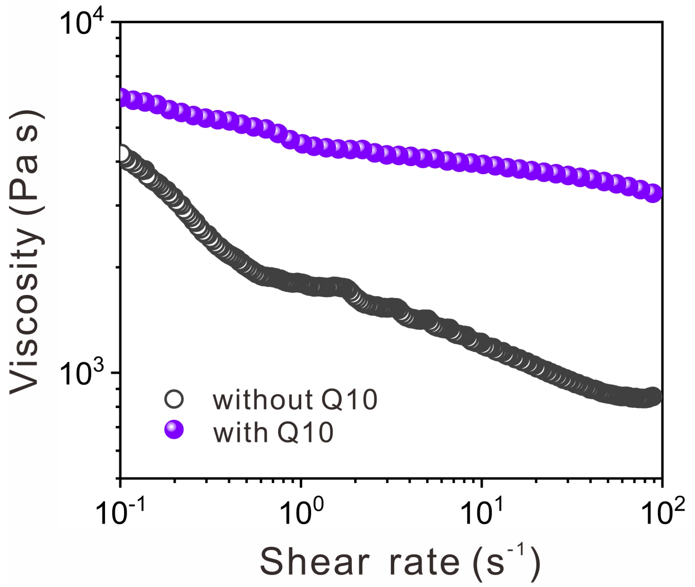

Supplementary Figure 23. The viscosity of different printing inks with varying shear rates. This comparison shows that adding Q10 (3 wt.%) significantly improves the viscosity of printing ink comparing to the control ink. The viscosity of ink decreases from 6000 Pa•s to 4000 Pa•s when increasing shear rates from 0.1 s^{-1} to 100 s^{-1}. However, without Q10, the viscosity decreases from 4000 Pa•s to 900 Pa•s at the same operating condition. This result indicates that the rheology property of printing ink can be easily tuned by adjusting the content of Q10 and printing speed, which is helpful for designing high-performance 3D TCHs.

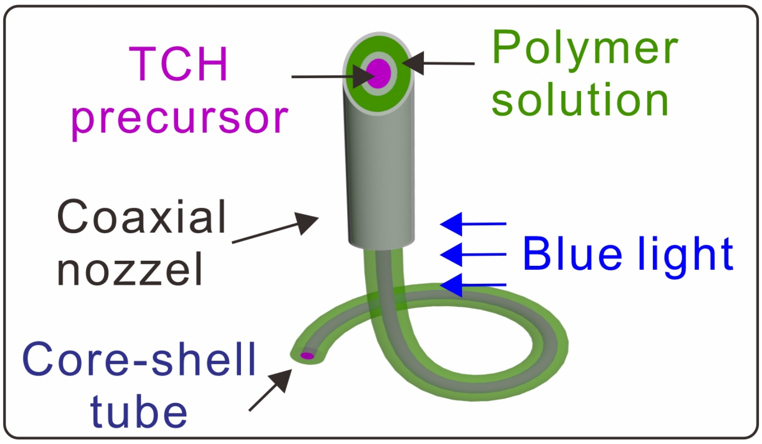

Supplementary Figure 24. Schematic illustration of co-axial 3D extrusion printing for free-standing helical TCHs. As shown in this figure, a co-axial nozzle (16G/12G) was used instead of the single one to print free-standing helical TCHs. Guest polymer solution and TCH precursor were injected into the outer and inner channels, respectively. When extruding them from the nozzle, the TCH precursor was rapidly photogelated, and the guest polymer was solidified according to a solvent-evaporation process. Therefore, the solvent used for dissolving polymers in the outer channel should have a low-boiling point, such as dichloromethane (~40 ºC), acetone (~56.6 ºC), and so on.

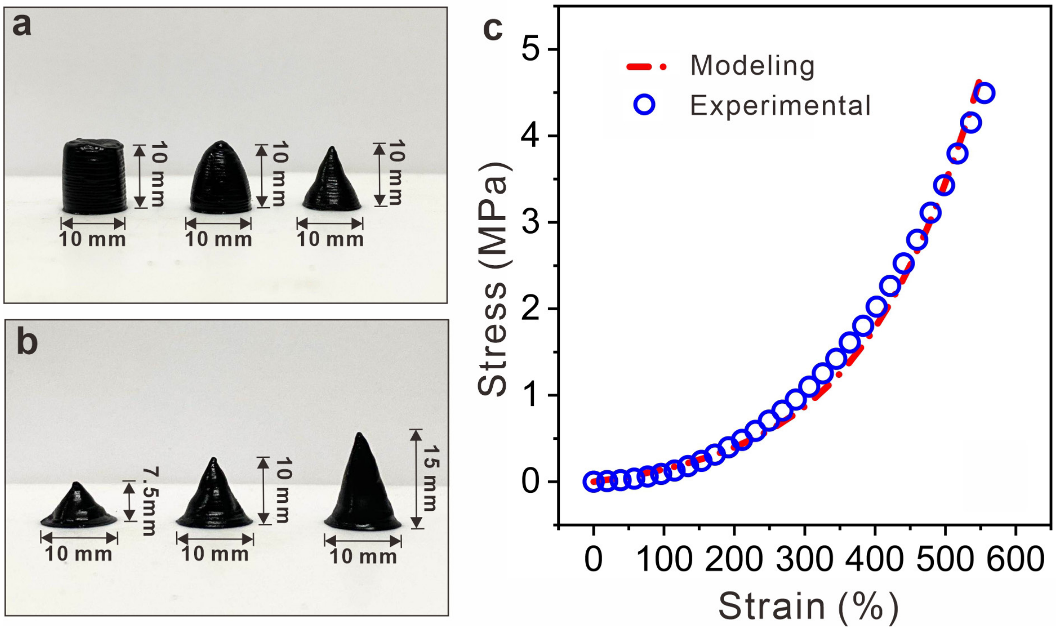

Supplementary Figure 25. 3D-printing and finite element analysis of TCHs with different geometries. (**a**) cylinder-, truncated- and cone-liked TCHs with the same diameter and height. (**b**) Cone-liked TCHs with the same diameter and varying heights. (**c**) The true stress-strain curves, respectively, were measured by the printed tensile sample and predicted by the Arruda-Boyce eight chain model.

Supplementary Note 3

The Arruda-Boyce eight chain model was used to model the hyperelasticity behaviors of printed TCHs. To simplify our discussion, we neglect the water absorption and dehydration of the hydrogel and focus on the elastic deformation. Therefore, the hydrogel is modeled as approximately incompressible materials, and the Cauchy stress $\mathbf{T}$ of the hydrogel is,

$$\mathbf{T} = \frac{\mu}{3J} \frac{\sqrt{N}}{\lambda_{\text{chain}}} \mathsf{L}^{-1}\left(\frac{\lambda_{\text{chain}}}{\sqrt{N}}\right) \overline{\mathbf{b}}' + K(J-1)\mathbf{I},$$

where L^{-1} is the inverse Langevin function with $\mathsf{L}(x) = \coth x - 1/x$, μ the shear modulus, K the bulk modulus, N the number of Kuhn segments between two crosslink sites, and λ_{chain} the effective stretch on each chain $\lambda_{\text{chain}} = \sqrt{tr(\overline{\mathbf{b}})/3}$. The deviatoric part of the isochoric left Cauchy-Green tensor is given by $\overline{\mathbf{b}}' = \overline{\mathbf{b}} - \frac{1}{3} tr(\overline{\mathbf{b}})\mathbf{I}$, where $\overline{\mathbf{b}} = \overline{\mathbf{F}} \cdot \overline{\mathbf{F}}^{\text{T}}$, and $\overline{\mathbf{F}} = J^{-1/3} \cdot \mathbf{F}$. As shown in Supplementary Fig. 25c, the Arruda-Boyce eight chain model can well predict the true stress-strain response of the printed hydrogel tensile samples by using the parameters of the initial shear modulus $\mu=0.038$ MPa, the bulk modulus K=2.65 Mpa, and the limiting network stretch $\sqrt{N} = 3.20$.

Since the printed structure has rotational symmetry, we used the 4-node bilinear axisymmetric quadrilateral, reduced integration, hourglass control element CAX4R in the commercial finite element software ABAQUS, and applied rotational symmetric boundary conditions to model the compression response of the printed structures. The friction coefficient between the printed samples and the fixture was set to be 0.08. The modeled contour plots of von Mises stress and the force-displacement curves were plotted in Figures 4g and h.

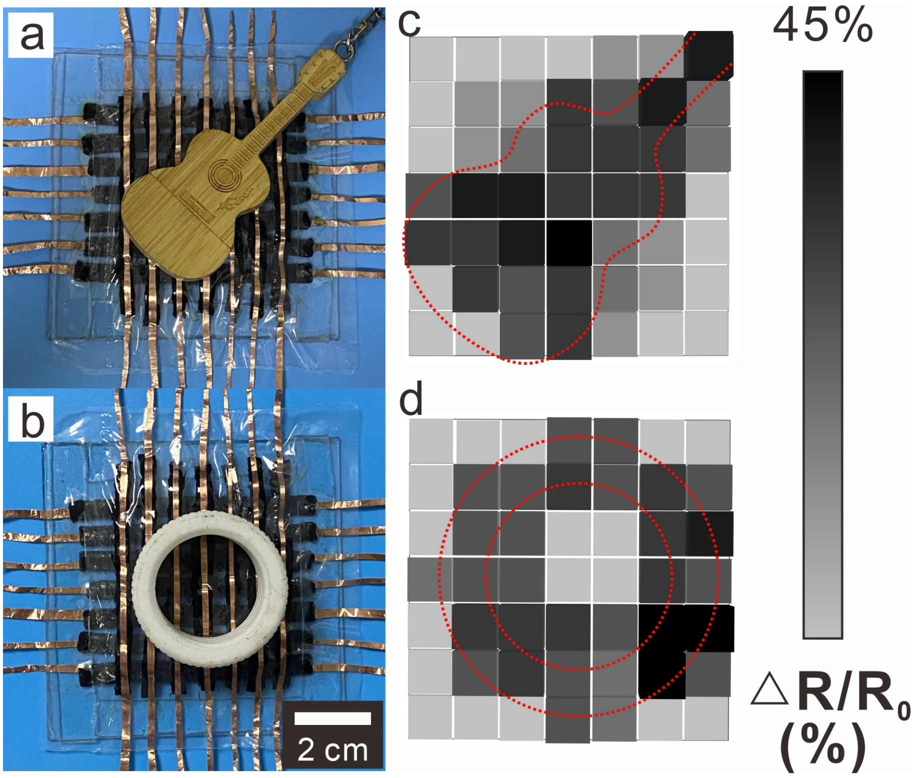

Supplementary Figure 26. A pressure sensor prepared by cone-like TCH ribbons for recognizing cargoes with different geometries. Photographs of pressure sensor with (**a**) piano-shaped USB and (**b**) plastic ring on its top. Prism map of the pressure sensor for (**c**) piano-shaped USB and (**d**) plastic ring. As shown in Figures (**a**) and (**b**), the printed TCH ribbons were perpendicularly fixed and assembled to a pressure sensor. All cone-liked 3D TCHs had tightly contacted each other, and the resistance of every contacting point was precisely measured by copper ribbons. To achieve the recognition of cargoes, the resistances of each unit was firstly measured before and after loading these cargoes. By comparing resistances during this process, the change of resistance and its distribution was easily obtained. Finally, the geometries of cargoes were obtained (Figures **c** and **d**).

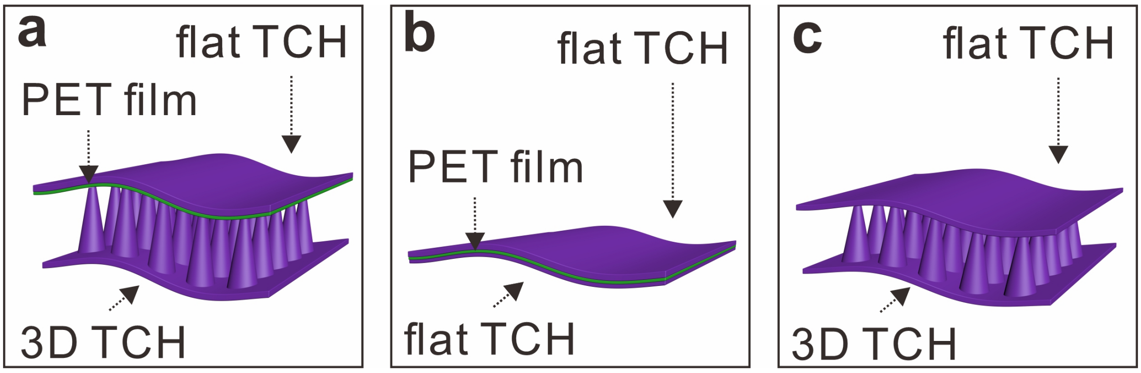

Supplementary Figure 27. Schematic illustration of three kinds of TCH-based pressure sensors. 3D and flat TCHs were prepared by the extrusion 3D printing technique. After that, these TCHs were assembled to (**a** and **b**) 3D- and flat-capacitor and (**c**) resistance sensors. For capacitors, a non-conductive PET film should be used to separate TCH electrodes. The thicknesses of flat TCHs and PET films were 800 μm and 50 μm, respectively. It is worthy of note that the geometry shape of 3D TCHs in this device was the same as that optimized in Supplementary Figure 24. Considering specific applications in the following study, the radius and height of cone-liked TCHs were reduced to half their original sizes. When detecting human motions, these devices were fixed on the body by thin, flexible 3M tapes.

Supplementary Table 1. Typically reported strategies for preparing conductive hydrogels[1, 2, 4-20]

Components	Method (Time)	Tensile strain (Stress/kPa)	Toughness (kJ/m^3)	2D patterns /3D structures	Conductivity	Anti-freezing (°C)	Patterning or printing
PEDOT:PSS /MC/κCA	mixed (>24 h)	N.M.	N.M.	3D (100 μm)	0.3 S/m	NO	Extruding
PEDOT:PSS /PAA	mixed (>36 h)	1(250)	125	2D (100 μm)	23 S/m	NO	Extruding
PEDOT/PPy	Mg^{2+} (>24 h)	NO	NO	N.M.	N.M.	NO	NO
PEDOT:PSS /PVA	mixed (~10 min)	7(2000)	7000	2D (> 1 cm)	10 kΩ/cm	-40 °C	Molding
PEDOT:PSS /PAAm	mixed (>72 h)	3.5(30)	53	N.M.	1.0 S/m	N.M.	NO
PEDOT:PSS /HPU	mixed (>48 h)	6(2500)	7500	2D (100 μm)	10 Ω/□	N.M.	Extruding
PEDOT:PSS /DBSA	mixed (~10 min)	NO	NO	2D (> 1 cm)	100 S/m	NO	Injection
PEDOT:PSS	Cu^{2+} (~30 min)	NO	NO	2D (100 μm)	N.M.	NO	Electrochemical patterning
PEDOT:PSS	mixed (>72 h)	NO	NO	2D (100μm) 3D (100 μm)	6000~ 15000 S/m	N.M.	Extruding
PEDOT:PSS	H_2SO_4 (>12 h)	NO	N.M.	2D (> 1 cm)	50~900 S/m	NO	Molding
EDOT/Agarose	Electro-P (~10 min)	NO	NO	2D (100 μm)	11 kΩ/□	NO	Electrochemical patterning
EDOT/Guar gum	APS/Fe^{2+} (~24 h)	NO	NO	N.M.	13 S/m	NO	NO
EDOT:PSS /HA-ALD/GC	APS/Fe^{2+} (48~72 h)	N.M.	N.M.	2D (> 1 cm)	3 S/m	NO	Extruding
EDOT/BC-*g*-PSS	APS (~72 h)	NO	N.M.	N.M.	24 S/m	NO	NO
EDOT/PEG	Fe-tosylate (>100 h)	N.M.	N.M.	2D (5 μm)	0.3 S/m	N.M.	Templating
EDOT/PVA/SA	APS (>72 h)	6(2500)	7500	N.M.	2.5 S/m	N.M.	NO
EDOT/PVA	APS/Fe^{3+} (>36 h)	4.5(600)	1350	N.M.	90 S/m	NO	NO
**EDOT/PVA-Ph**	**Ru(II)/APS (~30 min)**	**6(700)**	**2250**	**3D (~100 μm)**	**1~50 S/m**	**-36 °C**	**Extruding**

N.M. : Not Mentioned; **Electro-P :** Electro-Polymerization

Supplementary Table 2. Fabrication conditions of extruding printing for designing 3D structures

Structure	Nozzle size	Printing speed (mm s-1)	Applied pressure (MPa)
Straight lines	30G	2	0.5
Letters	30G, 23G, 21G	2, 5, 7	0.5,0.4,0.1
Folded, knotted, and spiral lines	25G	5	0.4
World map	23G	10	0.4
3D spiral coil	22G	10	0.3
3D pyramid	22G	10	0.3
Cylinder-, truncated- and cone-liked structure	22G	5	0.2
Stacked mesh	22G	5	0.2
TCH array with cone-liked units	22G	5	0.2
Bilayer TCH	16G/12G	5	inner:0.1<br>outer:0.5

References

1. Lu, B., Yuk, H., *et al.* Pure PEDOT:PSS hydrogels. *Nat. Commun.* **10,** 1043 (2019).

2. Yuk, H., Lu, B., *et al.* 3D printing of conducting polymers. *Nat. Commun.* **11,** 1604 (2020).

3. Guo, Y.-C., Cai, C. & Zhang, Y.-H. Observation of conformational changes in ethylene glycol–water complexes by FTIR–ATR spectroscopy and computational studies. *AIP Adv.* **8,** 055308 (2018).

4. Chen, Q., Lu, H., *et al.* Supramolecular Hydrogels for High-Voltage and Neutral-pH Flexible Supercapacitors. *ACS Appl. Energy Mater.* **1,** 4261-4268 (2018).

5. del Agua, I., Mantione, D., *et al.* Conducting Polymer Iongels Based on PEDOT and Guar Gum. *ACS Macro Lett.* **6,** 473-478 (2017).

6. Feig, V. R., Tran, H., *et al.* Mechanically tunable conductive interpenetrating network hydrogels that mimic the elastic moduli of biological tissue. *Nat. Commun.* **9,** 2740 (2018).

7. Feig, V. R., Tran, H., *et al.* An Electrochemical Gelation Method for Patterning Conductive

PEDOT:PSS Hydrogels. *Adv. Mater.* **31,** 1902869 (2019).
8. Gong, H. Y., Park, J.*, et al.* A Novel Conductive and Micropatterned PEG-Based Hydrogel Enabling the Topographical and Electrical Stimulation of Myoblasts. *ACS Appl. Mater. Interfaces* **11,** 47695-47706 (2019).
9. Inoue, A., Yuk, H.*, et al.* Strong adhesion of wet conducting polymers on diverse substrates. *Sci. Adv.* **6,** eaay5394 (2020).
10. Lee, Y.-Y., Kang, H.-Y.*, et al.* A Strain-Insensitive Stretchable Electronic Conductor: PEDOT:PSS/Acrylamide Organogels. *Adv. Mater.* **28,** 1636-1643 (2016).
11. Naficy, S., Oveissi, F.*, et al.* Printed, Flexible pH Sensor Hydrogels for Wet Environments. *Adv. Mater. Technol.* **3,** 1800137 (2018).
12. Qian, C., Higashigaki, T.*, et al.* Anisotropic Conductive Hydrogels with High Water Content. *ACS Appl. Mater. Interfaces* **12,** 27518-27525 (2020).
13. Rastin, H., Zhang, B.*, et al.* 3D printing of cell-laden electroconductive bioinks for tissue engineering applications. *J. Mater. Chem. B* **8,** 5862-5876 (2020).
14. Rong, Q., Lei, W.*, et al.* Anti-freezing, Conductive Self-healing Organohydrogels with Stable Strain-Sensitivity at Subzero Temperatures. *Angew. Chem. Int. Ed.* **56,** 14159-14163 (2017).
15. Sekine, S., Ido, Y.*, et al.* Conducting Polymer Electrodes Printed on Hydrogel. *J. Am. Chem. Soc.* **132,** 13174-13175 (2010).
16. Wang, S., Guo, G.*, et al.* Facile Soaking Strategy Toward Simultaneously Enhanced Conductivity and Toughness of Self-Healing Composite Hydrogels Through Constructing Multiple Noncovalent Interactions. *ACS Appl. Mater. Interfaces* **10,** 19133-19142 (2018).
17. Xu, Y., Patsis, P. A.*, et al.* Cytocompatible, Injectable, and Electroconductive Soft Adhesives with Hybrid Covalent/Noncovalent Dynamic Network. *Adv. Sci.* **6,** 1802077 (2019).
18. Yao, B., Wang, H.*, et al.* Ultrahigh-Conductivity Polymer Hydrogels with Arbitrary Structures. *Adv. Mater.* **29,** 1700974 (2017).
19. Yu, C., Wang, C.*, et al.* A Cytocompatible Robust Hybrid Conducting Polymer Hydrogel for Use in a Magnesium Battery. *Adv. Mater.* **28,** 9349-9355 (2016).
20. Zhang, S., Chen, Y.*, et al.* Room-Temperature-Formed PEDOT:PSS Hydrogels Enable Injectable, Soft, and Healable Organic Bioelectronics. *Adv. Mater.* **32,** 1904752 (2020).
