# Supplementary material for: Orthogonal photochemistry-assisted printing of 3D tough and stretchable conductive hydrogels
Source: Nat Commun. 2021 Apr 7;12:2082. doi: 10.1038/s41467-021-21869-y (PMC8027177; doi:10.1038/s41467-021-21869-y)
Supplement: Supplementary file 2 — Description of Additional Supplementary Files [file 41467_2021_21869_MOESM2_ESM.pdf]

Description of Additional Supplementary Files

File Name: Supplementary Movie 1
Description: Puncture experiment of TCH.

File Name: Supplementary Movie 2
Description: TCH-based actuator.
